# Two‐Stage Catalytic Conversion of Carbon Dioxide Into Aromatics Via Methane

**DOI:** 10.1002/anie.202517563

**Published:** 2025-10-21

**Authors:** Josepha J. G. Kromwijk, Angela E. M. Melcherts, Luke de Jong, Jules F. van Leusden, Joris C. L. Janssens, Ramon Oord, Ward van der Stam, Matteo Monai, Bert M. Weckhuysen

**Affiliations:** ^1^ Inorganic Chemistry and Catalysis group, Institute for Sustainable and Circular Chemistry Utrecht University Universiteitsweg 99 Utrecht 3584 CG the Netherlands

**Keywords:** Aromatics, CO_2_ methanation, Methane dehydroaromatization, Operando spectroscopy, Process optimization

## Abstract

In the refinery of the future, the input shifts from crude oil to biomass, plastic, and CO_2_. Therefore, we need to find alternative routes to produce chemical building blocks, such as aromatics, which are used in products like, for example, fuels. In this study, we investigated a two‐stage route to produce benzene from CO_2_. In two sequential reactions, CO_2_ is first converted into methane over a Ni/TiO_2_ catalyst, and methane is further reacted to yield benzene using a Mo/ZSM‐5 catalyst via the methane dehydroaromatization (MDA) reaction. Through a combination of thermodynamic calculations and experiments, we found the goldilocks conditions for performing this two‐stage process. The unreacted CO_2_ and H_2_ from the first reaction extended the benzene production in the second reaction. Using a reaction mixture of CO_2_, H_2_, and CH_4_ resulted in benzene production of at least 72 h, by suppressing carbon growth on the catalyst surface. However, the concentration range in which CO_2_ and H_2_ can be added to the feed without losing benzene production is narrow, as we show with H_2_ fluctuation experiments. We demonstrate that the combination of CO_2_ methanation and MDA allows us to catalytically convert CO_2_ into benzene with an overall yield of 5%.

## Graphical abstract

The development of a two‐stage process for converting CO_2_ via CH_4_ to C_6_H_6_ using a Ni/TiO_2_ catalyst in stage 1 and a Mo/ZSM‐5 catalyst stage 2. Thermodynamic calculations as well as experimentally varying parameters, aided in finding the optimal reaction conditions and reducing the coke deposition in stage 2. Furthermore, operando spectroscopy was employed to understand the increased catalyst stability in stage 2.

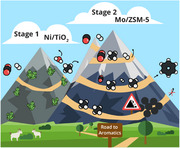

## Introduction

To achieve a carbon‐neutral economy by 2050, as laid out by the European Climate Law,^[^ ^1^ ^]^ our fossil‐based energy system must gradually transition to a system based on renewable alternatives, such as solar and wind. Similarly, the chemical industry has to transition to more sustainable feedstocks and energy input. The majority of the current greenhouse gas emissions of the petrochemical sector are caused by the operation and downstream of crude oil refineries. As laid out in a recent perspective by Vogt and Weckhuysen,^[^ ^2^ ^]^ in the so‐called refinery of the future, the carbon input would shift from crude oil to a mixture of carbon dioxide (CO_2_), biomass, and plastic. However, the exact moment of this transition may change depending on the political and economic situation.

**Figure 1:**
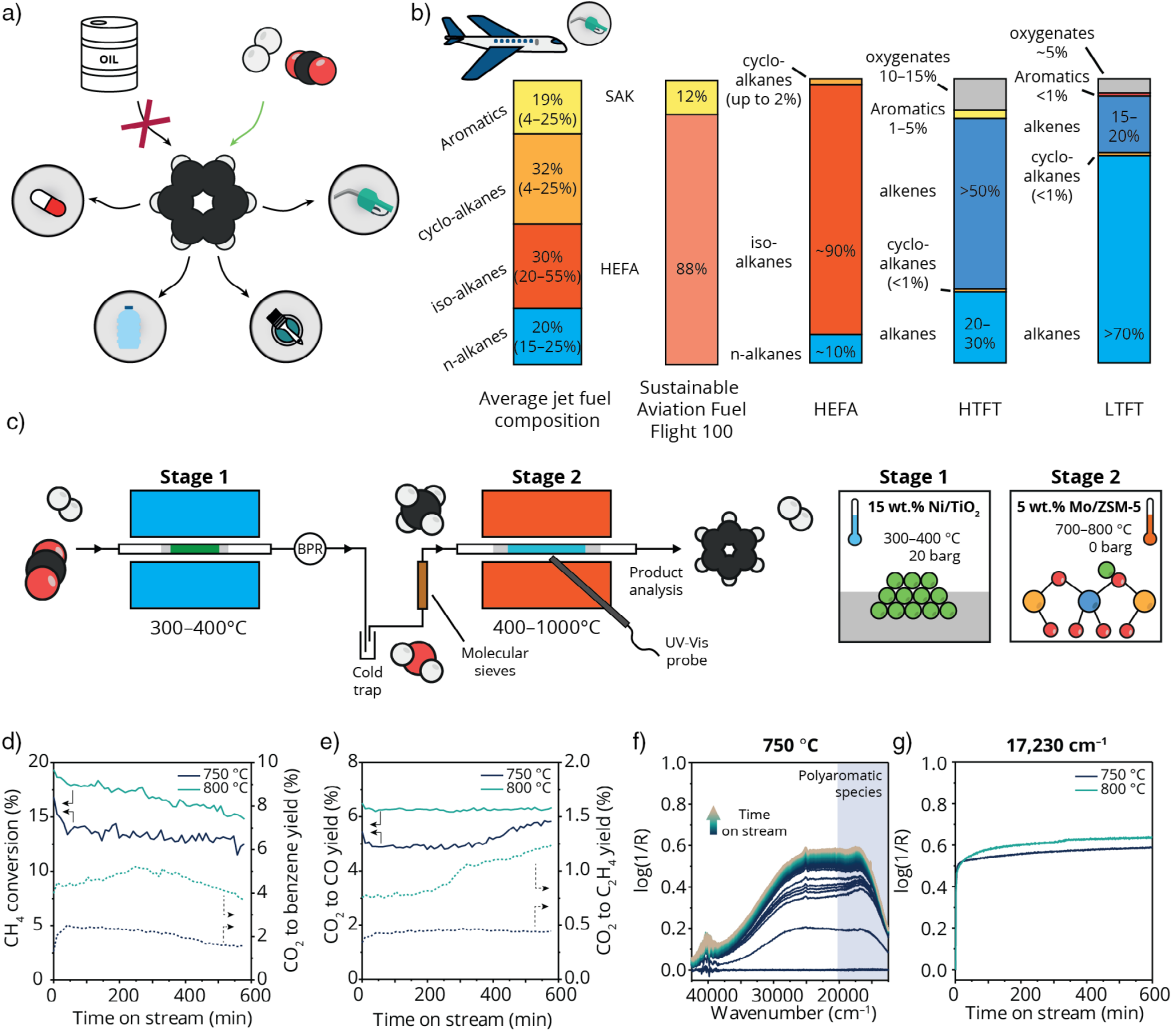
Concept of the two‐stage conversion of CO_2_ to aromatics via methane a) Schematic representation of the different products that can be made from benzene. b) Composition of an average jet fuel,^[^ ^6^ ^]^ compared to the composition of the fuel of Virgin Atlantic's first transatlantic flight composed of hydroprocessed esters and fatty acids (HEFA) and synthetic aromatic kerosene^[^ ^7^ ^]^ (SAK), HEFA,^[^ ^8^ ^]^ and high temperature^[^ ^9^ ^]^ (HTFT) and low temperature Fischer Tropsch synthesis^[^ ^9^ ^]^ (LTFT). c) Schematic representation of our lab‐built reactor system for the two‐stage conversion of CO_2_ into benzene via methane. A 15 wt.% Ni/TiO_2_ catalyst was used for CO_2_ methanation in stage 1 (CO_2_:H_2_ = 1:4, 400 °C, 20 barg), while in stage 2 a 5 wt.% Mo/ZSM‐5 catalyst was used for the methane dehydroaromatization (MDA) reaction (750 °C or 800 °C, 1 atm). A back‐pressure regulator (BPR) was used to pressurize stage 1 and a cold trap and molecular sieves after stage 1 were used to remove water from the MDA input stream. d)–f) Catalytic data of the two‐stage conversion of CO_2_ to benzene. d) CH_4_ conversion at stage 2 and the total benzene yield from CO_2_. e) Total CO and ethylene yield from CO_2_ in two‐stage process. F) Coke deposition on the MDA catalyst during the two‐stage reaction, as studied by operando UV–vis spectroscopy at 750 °C, with the blue region indicating polyaromatic carbon species, and G) the corresponding log(1/R) at 17 230 cm^−1^ over time. A fast darkening of the catalyst followed by a slow increase in absorbance indicates suppressed coking with respect to MDA using pure methane as feedstock.

The current output of a crude oil refinery consists of transportation fuels (81%) and chemicals (19%).^[^ ^2^ ^]^ Since transport electrification is expected to reduce the demand for hydrocarbons as fuels, in the future there should be a shift toward chemicals. A key example of important chemicals is aromatics, such as benzene, toluene, and xylene (BTX). Aromatics can be found in plastics, solvents, detergents, insecticides, and pharmaceuticals (Figure 1a).^[^ ^3^ ^]^ In 2023, the aromatics market size was valued at 25 billion US dollars,^[^ ^4^ ^]^ and is expected to grow to 39 billion US dollars by 2032. Currently, the production of aromatics is crude oil‐based, as ∼ 70% of BTX is obtained from steam‐reforming naphtha.^[^ ^5^ ^]^ Therefore, when the input of the refinery shifts from crude oil to CO_2_, biomass, and plastic, and the aromatics market keeps expanding, it is essential to find a new route toward aromatics.

The essence of finding new production routes toward aromatics becomes evident when looking at the example of the transition to sustainable aviation fuels (SAF) in the aerospace industry. The typical composition of jet fuel is a combination of paraffins and 4%–25% aromatics.^[^ ^6^, ^10^, ^11^ ^]^ Technologies to produce SAF, such as hydroprocessed esters and fatty acids (HEFA) and fischer tropsch synthesis (FTS), only provide a part of the fuel, as these processes result in linear and branched hydrocarbons.^[^ ^6^, ^11^, ^12^, ^13^ ^]^ Airplanes still require aromatics for safe flight operation, so in this case, paraffinic SAF must be blended with aromatics.^[^ ^14^, ^15^ ^]^ This was demonstrated in 2023 when the Virgin Atlantic Flight100 was the first transatlantic flight powered by 100% SAF. The SAF consisted of a blend of 88% HEFA and 12% synthetic aromatic kerosene (SAK), derived from plant sugars (Figure 1b).^[^ ^12^ ^]^

Producing aromatics from biomass and plastic waste has already been explored in literature.^[^ ^16^, ^17^, ^18^, ^19^, ^20^, ^21^ ^]^ The advantage of using biomass as a starting material to produce aromatics is its high aromatic and low oxygen content. However, biomass, more specifically lignin, contains structurally complex molecules, making it demanding to process. Another major challenge in the application of depolymerization of biomass toward aromatics is efficient separation and purification.^[^ ^22^, ^23^ ^]^ Nonetheless, the production of biomass‐derived aromatics emits 42% less greenhouse gas emissions compared to the fossil fuel equivalent, making it a promising route.^[^ ^24^ ^]^ Converting plastic waste into aromatics, on the other hand, results in a 12% reduction in greenhouse gases compared to its fossil fuel equivalent.^[^ ^24^ ^]^ The production of aromatics from plastic waste is more sustainable than incineration combined with energy recovery. An example of this approach is currently being explored by BioBTX,^[^ ^25^ ^]^ a company located in the Netherlands, which is already doing pilot‐scale experiments using plastic waste and biomass as sources to produce BTX.

Another method to produce fossil‐free aromatics is from CO_2_, either captured from point sources (e.g., metallurgical blast furnaces or cement industries) or directly from the air. CO_2_ hydrogenation with H_2_ produced in electrochemical processes, powered by solar panels and/or wind turbines, is a way to obtain valuable chemicals. However, suppressing hydrogenation activity to produce C─C coupled products from CO_2_ is one of the biggest challenges within the field of catalytically activating small molecules. A promising strategy to produce more complex chemicals from CO_2_ is through combining catalytic processes.^[^ ^26^ ^]^ By first converting CO_2_ into a platform molecule, such as methanol, syngas, or methane, C─C coupled products can be generated in a second reactor. To produce aromatics specifically, methanol or methane are the most suitable intermediate molecules.^[^ ^27^, ^28^ ^]^ The use of CO_2_ instead of methane directly has the additional benefit of potential emission reductions and a decrease in feedstock prices which could make the overall process worthwhile.^[^ ^28^ ^]^ The conversion of methanol to aromatics (MTA) is typically carried out over Zn‐ and Ga‐based ZSM‐5 catalysts.^[^ ^27^ ^]^ A direct approach via a tandem CO_2_‐to‐methanol/MTA catalyst Zn_0.1_Ti_0.9_O_x_/HZSM‐5 by Zhou et al. showed promising results in terms of stability, as they managed to produce aromatics for 100 h on stream.^[^ ^29^ ^]^ Using bifunctional catalysts is however, thermodynamically challenging: both CO_2_ hydrogenation and methanol synthesis are highly exothermic, making it a thermodynamic mismatch with the endothermic aromatic formation. The separation of the processes in two catalytic systems also has the advantage that there is more flexibility to tune each step individually to increase conversion and BTX selectivity.

Miller et al. compared methanol to methane as intermediates to benzene and showed that both have their own advantages and disadvantages.^[^ ^28^ ^]^ The reaction to convert CH_4_ into aromatics, known as the methane dehydroaromatization (MDA) process, has gained interest due to its high selectivity.^[^ ^28^ ^]^ Producing aromatics from CO_2_ via CH_4_ has been reported in the literature by Zhu et al.^[^ ^30^ ^]^ For this tandem reaction, Zhu et al. reported a Ni/SiO_2_ catalyst for CO_2_ methanation combined with a Mo/ZSM‐5 catalyst to convert CH_4_ into aromatics in the MDA process. Although this work shows the feasibility of this process, the benzene yield from CO_2_ of 1.7 × 10^−3^ can be improved, and effective CH_4_ recycling could be implemented to increase the overall yield.^[^ ^28^ ^]^ Because of the flexibility of a two‐stage system, both catalytic systems can also be optimized to further increase benzene yield. In the first stage, full conversion of CO_2_ to methane is difficult, so residual CO_2_ and H_2_ are still in the gas stream to the MDA reaction in stage 2. Typically, fast catalyst deactivation is observed for the MDA reaction since coke formation is thermodynamically favorable. From the literature, we know CO_2_ and H_2_ can be beneficial in extending the MDA catalyst lifetime as they are known to suppress coke formation.^[^ ^31^, ^32^, ^33^, ^34^, ^35^, ^36^, ^37^, ^38^, ^39^, ^40^ ^]^

In this work, we investigate the combination of the CO_2_ methanation reaction with the methane dehydroaromatization reaction to produce benzene. An integrated reactor system was developed in which a 15 wt.% Ni/TiO_2_ catalyst for the CO_2_ methanation and a 5 wt.% Mo/ZSM‐5 zeolite catalyst were investigated for the two‐stage process. To monitor the coke deposition catalyst surface, optical fiber probes were installed. Using a combination of thermodynamic calculations and experimentally varying reaction conditions, we were able to determine the boundaries of this system. To mitigate the effect water can have on the zeolite catalyst for the MDA reaction we removed the water formed during the CO_2_ methanation reaction between the reactors. Furthermore, we show it is essential to reach CH_4_ yields of at least 93% in the first reaction since otherwise residual CO_2_ and H_2_ inhibit the formation of benzene in the MDA stage. Nonetheless, small amounts of CO_2_ and H_2_ in the CH_4_ feed prevent the deactivation of the MDA catalyst by limiting coke formation on the catalyst surface, as observed with optical fibers during operation. We show that these concentration ranges of CO_2_ and H_2_ are narrow, as fluctuating H_2_ input can be detrimental to the benzene production. The route presented could be a starting point for the development of alternative routes for benzene production.

## Results and Discussion

### Two‐Stage Conversion of CO_2_ to Benzene via Methane

To enable the two‐stage conversion of CO_2_ to benzene via CH_4_, an integrated reactor system equipped with optical fibers was developed, which is schematically shown in Figure 1c. The lab‐scale reactor consists of three consecutive ovens (Figures S1, S2). The first reactor oven, for CO_2_ methanation, can be heated up to 1000 °C when operating at atmospheric pressure and using quartz reactors, or up to 600 °C and 30 barg for steel reactors (Figure S2B,C). Next to oven 1 is an intermediate oven with a back‐pressure regulator (BPR) to pressurize the first reactor, and valves to switch the gas output from reactor 1 to either the gas chromatograph or the second reactor oven. The second reactor oven, for the methane dehydroaromatization reaction, consists of three temperature zones that can be heated separately up to 1000 °C, which can be used to simulate thermal gradients in industrial reactors (Figure S2FG). The first and second reactor ovens have separate mass flow control (MFC) systems equipped with He, N_2_, O_2_, CO_2_, H_2_, CH_4_, and CO, and a separate outlet to the GC, allowing for modular operation. Moreover, both reactor ovens have an opening suited for the application of optical fiber probes for performing operando spectroscopy (technical drawings in Supporting Information). When operating in two‐stage configuration, an ethylene glycol cold trap set to 5 °C and molecular sieves were placed between the BPR and the third reactor to ensure water formed in the first reaction to rule out the effects of water (Figure S2D,E).

For the two‐stage process, a Ni/TiO_2_ catalyst was selected for the CO_2_ methanation reaction. Supported nickel catalysts are generally considered for CO_2_ methanation reactions due to their high hydrogenation activity and cost‐effectiveness compared to other noble metal hydrogenation catalysts.^[^ ^41^ ^]^ Furthermore, titania‐supported nickel catalysts show high selectivity toward CH_4_ compared to other supports, like SiO_2_ or Al_2_O_3_.^[^ ^42^, ^43^ ^]^ For the methane dehydroaromatization (MDA) reaction in the second stage, a Mo/ZSM‐5 catalyst was used in view of its superior performance compared to other catalytic systems.^[^ ^44^, ^45^ ^]^ More details on the chosen catalyst systems and the active sites can be found in the Supporting Information.

During the two‐stage process, a 15 wt.% Ni/TiO_2_ catalyst (Figure S3, Table S4) was operated at 400 °C and 20 barg to achieve ∼ 95% CO_2_ conversion and 100% CH_4_ selectivity in the first stage. Full water removal was confirmed with a humidity sensor before passing the feed to the second stage. Here, the methane dehydroaromatization (MDA) was performed at 750 or 800 °C using a 5 wt.% Mo/ZSM‐5 catalyst (Table S4) to convert up to 20% of the initial CO_2_ into, amongst others, benzene, CO, and ethylene (Figures 1d,e, S4). Interestingly, for the two‐stage process, we observed a relatively constant benzene formation with time on stream, whereas at these high reaction temperatures, full deactivation of the MDA catalyst is typically observed within 10 h due to coke formation.^[^ ^46^ ^]^ This suggests that carbon deposition was suppressed in our two‐stage process. To study the coke formation on the Mo/ZSM‐5 catalyst during the two‐stage process, an optical fiber probe was used to perform operando UV–visible (UV–vis) spectroscopy.

Figure 1f shows the time evolution of the operando UV–vis spectra recorded during the reaction at 750 °C over 10 h. In general, an increase in absorbance over time was observed in regions corresponding to polyaromatic hydrocarbons (below 20 000 cm^−1^), hydrocarbon pool intermediates (35 000–30 000 cm^−1^), and neutral aromatics (38 000–35 000 cm^−1^).^[^ ^47^ ^]^ Additionally, some sharp features can be observed which are artifacts from the UV–vis light source. In literature, the deactivation mechanism of the MDA process is explained by the formation of polyaromatic coke species, which cannot leave the zeolite pore, and whose absorption region is indicated in blue in Figure 1f.^[^ ^46^ ^]^ To follow the formation of these species, the intensity of the band at 17 230 cm^−1^ was plotted as a function of time (Figure 1g). This absorption band showed a rapid increase in early time on stream, similar to regular MDA.^[^ ^47^ ^]^ However, the further growth of this band was suppressed over the 10 h of reaction, confirming that the gas composition from the first stage aids in extending the lifetime of the MDA catalyst by reducing coke formation. To explain the extended catalyst lifetime and test the boundaries of this two‐stage process, thermodynamic calculations and simulation experiments were performed.

### Exploring Thermodynamic Boundaries and Reaction Conditions

Based on CO_2_ and H_2_ cofeeding studies reported in literature, we hypothesized that coke suppression and increased stability of benzene production in our two‐stage process could be due to the presence of unreacted CO_2_ and H_2_ in the gas stream from stage 1. Indeed, CO_2_ has a stabilizing effect on the MDA catalyst activity by inducing methane reforming reactions, which leads to the formation of CO and H_2_; species that can suppress coke formation.^[^ ^36^, ^37^, ^38^, ^39^, ^40^ ^]^ Furthermore, cofeeding H_2_ can hydrogenate coke species.^[^ ^31^, ^32^, ^33^, ^34^, ^40^ ^]^ However, when excessive amounts of CO_2_ or H_2_ are present, the formation of aromatic products can be completely inhibited.^[^ ^35^ ^]^

**Figure 2:**
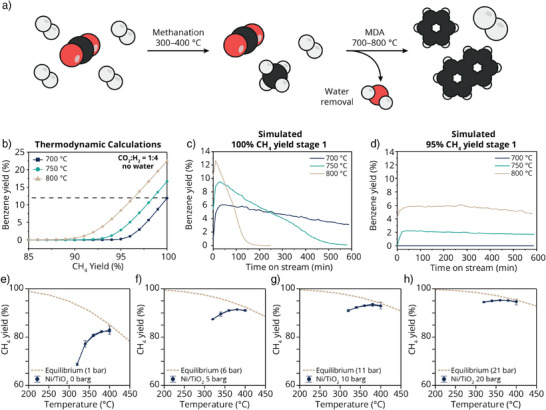
Exploring the thermodynamic boundaries of the two‐stage CO_2_ methanation and methane dehydroaromatization (MDA) reactions. a) Schematic representation of the two‐stage process with CO_2_ methanation in step 1 with a CO_2_:H_2_ ratio of 1:4, followed by MDA. Water is removed after the first step, but unreacted CO_2_ and H_2_ from the first stage will be present in the subsequent MDA reaction. b) Thermodynamic equilibrium calculations of the MDA reaction at 700, 750, and 800 °C considering CH_4_ yields between 85%and 100% in the CO_2_ methanation reaction using a CO_2_:H_2_ ratio of 1:4 and water removal. c)–d) Benzene yields based on CH_4_ input, obtained from catalytic performance testing of the MDA reaction over a 5 wt.% Mo/ZSM‐5 catalyst at 700, 750, and 800 °C using a reaction mixture that would be obtained from the CO_2_ methanation reaction in case of 100% and 95% CH_4_ yield using a CO_2_:H_2_ ratio of 1:4. e)–h) CH_4_ yield in catalytic CO_2_ methanation over a 15 wt.% Ni/TiO_2_ catalyst at 320–400 °C, going both up and down in temperature showing its stability after different temperature exposure, and different pressures (0, 5, 10, and 20 barg, for E to H respectively, blue data points). The brown dashed lines represent the thermodynamic equilibrium CH_4_ yield at each pressure. The error bars are from the standard deviation of the GC areas obtained during 90 min isotherms.

To understand the boundaries of our two‐stage process, schematically depicted in Figure 2a, we carried out thermodynamic equilibrium calculations using the HSC Chemistry 9.1 Gem equilibrium composition module (see Supporting Information for details on the calculations). First, we looked into the effect of the H_2_O formed in stage 1 on the benzene production in stage 2. Figure S8A shows the thermodynamic benzene yield in stage 2 at 750 °C as a function of varying CH_4_ yield in stage 1, where a 1:4 CO_2_:H_2_ was applied. It shows that the H_2_O formed in stage 1 completely suppresses the benzene formation in stage 2. Although literature shows small amounts of H_2_O could be beneficial for coke suppression^[^ ^48^, ^49^ ^]^ we chose to completely remove H_2_O to ensure possible benzene production and rule out its possible effect on the benzene yield and catalyst stability. Furthermore, we considered various CO_2_:H_2_ ratios as input for the CO_2_ methanation reaction, from slightly below to slightly above stoichiometric: 1:3.2, 1:4, and 1:6. Only a molar ratio for CO_2_:H_2_ of 1:4 makes it thermodynamically possible to form benzene (Figure S8B). Figure S8C shows that CO_2_ removal could be employed in future experiments as it thermodynamically leads to increased benzene yield. However, it should be investigated whether this outweighs the positive effects of CO_2_ addition on coke removal.

The benzene yields of the MDA reaction at 700, 750, and 800 °C were calculated as a function of CH_4_ yield and shown in Figure 2b, assuming an initial feed of CO_2_:H_2_ in 1:4, complete H_2_O removal, and a 100% selectivity toward CH_4_. This demonstrates the thermodynamic boundaries of the system; it is impossible to form benzene when the CH_4_ yield in the first reactor is lower than 90%. Moreover, the calculations show that carrying out the MDA reaction at increasingly higher reaction temperatures allows for operating the first reactor at lower CH_4_ yields. For instance, when a CH_4_ yield of 95% is achieved in stage 1, the MDA reaction should be performed at least at 750 °C, since at 700 °C it is thermodynamically not possible to form benzene.

Although higher MDA reaction temperatures seemingly result in higher benzene yields, this calculation does not account for coke formation. When carbon deposition is taken into account (Figure S8D), the equilibrium completely shifts to coke with increasing reaction temperature. Nevertheless, as mentioned before, the presence of CO_2_ and H_2_ hindered coke formation and allowed us to operate at elevated temperatures without dramatic deactivation. Another effect of lower CH_4_ yields, and thus higher amounts of CO_2_ and H_2_ in the feed, is that the selectivity toward CO and H_2_ increases due to the methane reforming reaction becoming more favorable (Figure S8E). Overall, these calculations emphasize that to achieve high aromatic yields, H_2_O should be removed, and a minimum of 90% CH_4_ yield should be achieved for the CO_2_ methanation reaction using a molar ratio of 1:4 for CO_2_ and H_2_.

To study the effect of CH_4_ yield in the CO_2_ methanation reaction on the MDA reaction, we performed MDA catalytic tests using gas streams simulating the gas composition which would come from the first reactor when 100%, 95%, and 93% CH_4_ yields are obtained, assuming complete H_2_O removal between the two reactors and 100% CH_4_ selectivity in stage 1 (gas mixtures are presented in Table S2). In Figure 2c, the benzene yields as a function of time on stream are shown for a simulated stream where 100% CH_4_ yield is reached in the first reactor, for different MDA reaction temperatures. All catalytic tests showed rather high initial activity, reaching a maximum of 6%, 10%, and 13% benzene yield for 700, 750, and 800 °C, followed by deactivation. The temperature had a significant impact on the deactivation rate; at 700 °C, the benzene yield slowly dropped over time, but after 10 h the catalyst still showed benzene production (∼ 3% yield). At 750 and 800 °C, the catalysts were fully deactivated after 500 and 150 min on stream, respectively. Both the increased initial yield and coke formation at higher reaction temperatures are in line with our thermodynamic calculations.

The picture changes when a gas mixture simulating 95% CH_4_ yield in stage 1 is used (Figure 2d). As predicted by thermodynamics, the benzene production was completely suppressed when performing the MDA reaction at 700 °C. At 750 and 800 °C, benzene was still produced, albeit at lower yields compared to the case of 100% CH_4_ yield. Interestingly, however, the benzene production remained almost constant for 10 h on stream. Besides benzene, CO was constantly produced when CO_2_ and H_2_ were present in the feed (Figure S9D–F), showing that parallel to the aromatization reaction, methane reforming reactions also take place. In the literature, the stabilizing role of CO_2_ and H_2_ on the MDA reaction has been discussed, although the exact reaction mechanism remains unclear. In the case of cofeeding CO_2_, it has been hypothesized that the CO formed during the dry methane reforming reaction (i.e., CH_4_ + CO_2_ → 2 CO + 2 H_2_) and reverse Boudouard reaction (i.e., CO_2_ + C → 2 CO) plays an important role. It has been suggested that the carbon atom of the formed CO ends up in the aromatic product, whereas the oxygen atom plays a role in the removal of surface coke.^[^ ^35^, ^36^, ^38^, ^39^, ^50^, ^51^ ^]^ The higher amount of CO present could also influence the nature of the active site. It is known that CO acts as a carburizing agent for molybdenum under these conditions.^[^ ^52^ ^]^ Therefore, it is likely that the active site becomes more carbidic in nature with increasing CO_2_, and thus, CO concentrations. However, operando X‐ray absorption spectroscopy (XAS) studies would be required to draw conclusions on this, which could be the focus of future studies.

When using a simulated stream resembling the case of 93% CH_4_ yield in the first reactor, benzene was no longer produced when performing the reaction at 750 °C (Figure S9I), which is again in line with thermodynamics. In this case, it would be most favorable to perform the reaction at 800 °C since we still observed substantial and constant benzene yields (Figure S9I). Nonetheless, increasingly higher CO_2_ concentrations in the feed result in higher CO selectivities (Figure S9D–F) and suppressed benzene production. Therefore, in the ideal case, the CO_2_ methanation is carried out at ∼ 95% CH_4_ yield, so a stable aromatic yield can be achieved, but reforming reactions are suppressed.

To determine the operational boundaries for the CO_2_ methanation reaction, thermodynamic equilibrium calculations and catalytic performance tests were done (Figure 2e–h). The CO_2_ methanation reaction is exothermic and, as shown by the thermodynamic equilibrium calculations, the reaction is favored at low temperatures and high pressures (dashed line in Figure 2e–h). To be able to achieve high CH_4_ selectivity, a 15 wt.% Ni/TiO_2_ was selected^[^ ^42^, ^43^ ^]^ and its performance was tested at 0, 5, 10, and 20 barg at temperatures between 320 and 400 °C (blue line in Figure 2e–h). At low reaction temperatures, the thermodynamic CH_4_ yield was not achieved. However, at high pressure and temperature, we experimentally achieved the thermodynamic limit for CH_4_ production. The small differences between experimental results and calculated equilibria could arise from slight temperature differences, as the thermocouple is placed outside the catalyst reactor. To achieve the desired 95% CH_4_ yield with the catalyst we use, it is essential to perform the CO_2_ methanation reaction at 400 °C and 20 barg.

### Understanding Improved MDA Catalyst Stability

**Figure 3:**
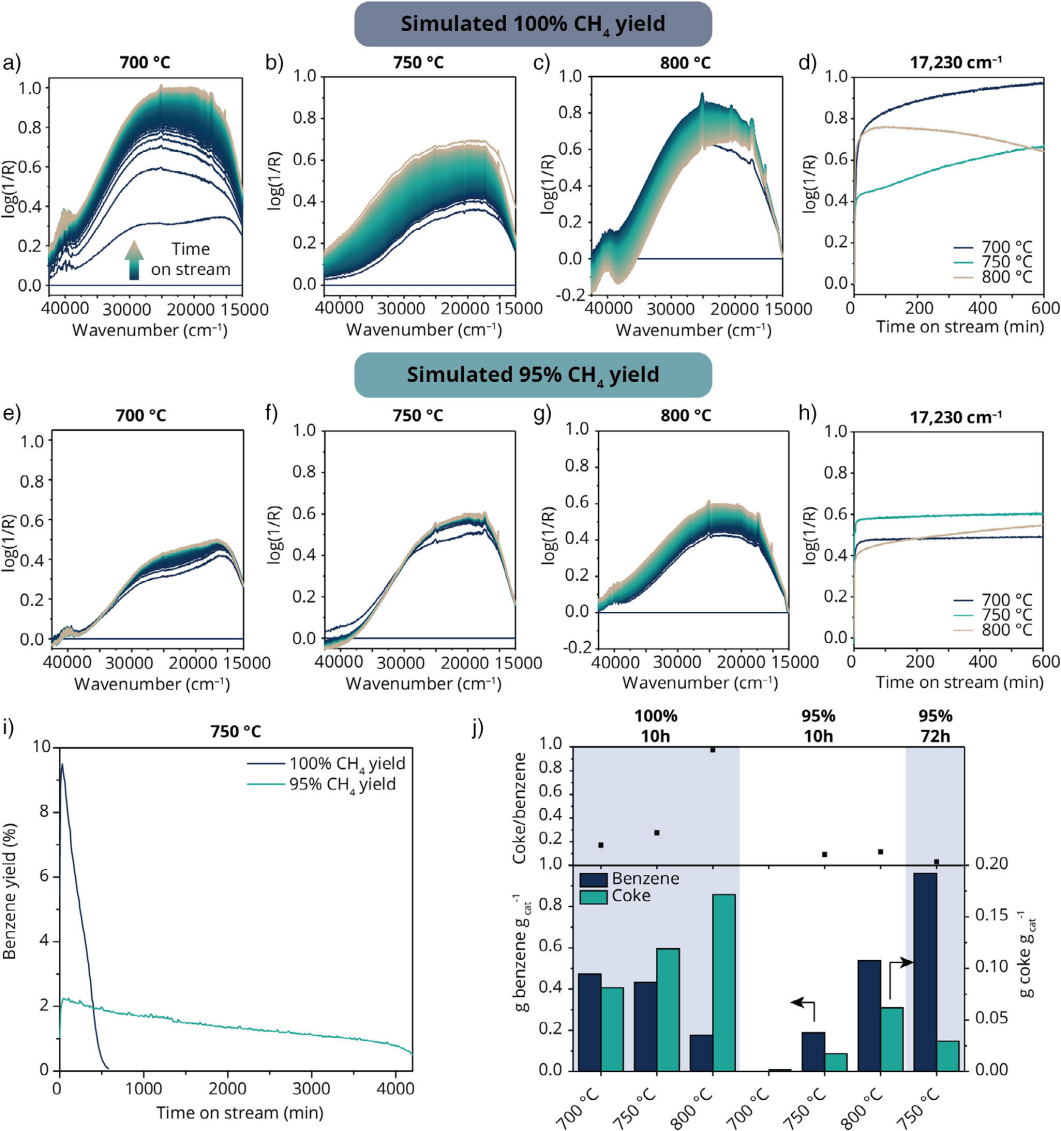
Showcasing the stability of the MDA catalyst at various simulated CH_4_ yields from stage 1. Operando UV–vis diffuse reflectance spectra recorded during the MDA reaction over 5 wt.% Mo/ZSM‐5 catalysts at 700, 750, and 800 °C at a)–c) simulated 100% and e)–g) 95% CH_4_ yield in the first reactor. D and H show the corresponding log(1/R) at 17 230 cm^−1^ over time for the MDA reactions carried out with simulated 100% and 95% CH_4_ yield at 700, 750, and 800 °C. i) Benzene yield for the MDA reaction at 750 °C at simulated 100% and 95% CH_4_ yield over 72 h. J) Coke and benzene amounts produced during the simulated two‐stage MDA tests, as determined from thermogravimetric analysis (TGA) on 5 wt.% Mo/ZSM‐5 catalysts and online gas chromatography (GC), respectively.

To understand the reason for improved catalyst stability of the second stage MDA reaction under incomplete CO_2_ conversion, operando UV–vis diffuse reflectance spectroscopy was carried out during MDA experiments in which various CH_4_ yields from stage 1 were simulated. In Figure 3a–c, the operando UV–vis diffuse reflectance spectra during the reaction at simulated 100% CH_4_ yield and 700, 750, and 800 °C are shown, respectively. Over time, we observe an increase in absorbance over the wavenumbers we measure (42 500–12 500 cm^−1^), which is comparable to what has been reported earlier in the literature for the MDA reaction.^[^ ^47^ ^]^ When a steam simulating 95% CH_4_ yield was used, the shape of the UV–vis diffuse reflectance spectra was similar, indicating similar coke species are present (Figure 3e–g). However, the band intensity grew faster initially and then stabilized over time. This becomes evident in Figure 3d,h, where the log(1/R) value for the absorption band at 17 230 cm^−1^, representative of the formation of polyaromatic hydrocarbons, was plotted as a function of time on stream. The plots show that in the case of simulated 100% CH_4_ yield, the 17 230 cm^−1^ absorption band continued growing over time, except for the experiment at 800 °C, where the log(1/R) value peaked at around 60 min and then decreased over time. We hypothesize this is due to a combination of two factors. First of all, at 800 °C, there is a higher amount of blackbody radiation compared to 700 and 750 °C. The second factor is the changing emissivity of the material as coke formation results in a blacker material over time.

Comparing the trends of the 100% CH_4_ yield change to the simulated 95% CH_4_ yield case, the initial growth in 17 230 cm^−1^ absorption band intensity was faster for the latter, but then stabilized with time on stream, and the trends are comparable to the two‐stage process (Figure S5). Such trends were also observed in the case when 93% CH_4_ yield was simulated (Figure S10). This indicates that CO_2_ and H_2_ prevent the accumulation of coke on the catalyst surface. To test the stability of the MDA catalysts over longer times, the MDA reaction was performed for 72 h at 750 °C using the simulated 100 and 95% CH_4_ yield conditions (Figure 3i). Over this time, a ∼ 50% drop in benzene production was observed for 95% CH_4_ yield, while the catalyst was fully deactivated in the first 8 h for the simulated 100% CH_4_ yield case, showing an improvement in catalyst stability for the 95% case.

The effects of cofeeding CO_2_ and H_2_ on the coke formation are also discernable when analyzing the spent samples with thermogravimetric analysis (TGA) (Figures S11, S12). For the experiments simulating 100% CH_4_ yield, the coke content increased with reaction temperature, whereas the total benzene production decreased due to faster catalyst deactivation (Figure 3j). In the 100% CH_4_ yield case, it is therefore preferred to carry out the reaction at 700 °C to achieve lower coke/benzene (C/B) ratios. The C/B ratio dropped from 1 to 0.1 when executing the reaction under simulated 95% CH_4_ yield and 800 °C, and the values were comparable to the two‐stage process (Figure S6). No data point is present for the reaction at 700 °C, because no benzene was produced. At higher reaction temperatures, however, cofeeding CO_2_ and H_2_ resulted in significantly less coke production with respect to benzene. The lowest coke/benzene ratio was observed when the reaction was performed for 72 h at 750 °C under the simulated 95% CH_4_ yield conditions. Compared to the 750 °C 100% CH_4_ yield case, twice the amount of benzene and less than 25% of the coke were produced. These results explain the MDA catalyst stability during our two‐stage process when the right conditions are used and also suggest the potential for applying this process at an industrial scale for longer times.

It is hypothesized that coke is not the only factor contributing to the deactivation of Mo/ZSM‐5 catalysts for the MDA reaction, but molybdenum sintering also plays a role.^[^ ^46^, ^53^, ^54^, ^55^ ^]^ Over the catalyst lifetime, the Mo species detach from their anchoring point and progressively migrate and sinter. Density functional theory (DFT) calculations suggest that Mo carbide particles with a C/Mo ratio greater than 1.5 prefer to anchor on external Si sites and thus tend to migrate to the external surface of the zeolite. The formation of MoC_x_ particles on the zeolite outer surface leads to faster catalyst deactivation since these species are more prone to coking.^[^ ^56^ ^]^ Cofeeding CO_2_ and H_2_ leads to an improvement in catalyst stability, but when examining the spent samples used in the simulation experiments as well as the two‐stage reaction with HAADF‐STEM, we observe molybdenum particles in all cases (Figure S7). Whether the catalyst is fully deactivated (in the simulated 100% CH_4_ yield case) or is still producing benzene after 72 h (in the simulated 95% CH_4_ yield case), these particles are present. This suggests that the role of large Mo clusters on catalyst deactivation is limited. However, the HAADF‐STEM images do not provide insights into the chemical nature of these particles, which could be different in the case of a fully deactivated catalyst compared to the one that is still active. Although these images do show smaller Mo clusters in the zeolite pores, we cannot conclude whether even smaller Mo sites are present, which could provide activity. To understand these structure–performance relationships better, operando X‐ray studies are required.

### Potential of Two‐Stage Process for CO_2_ Conversion to Aromatics

**Figure 4:**
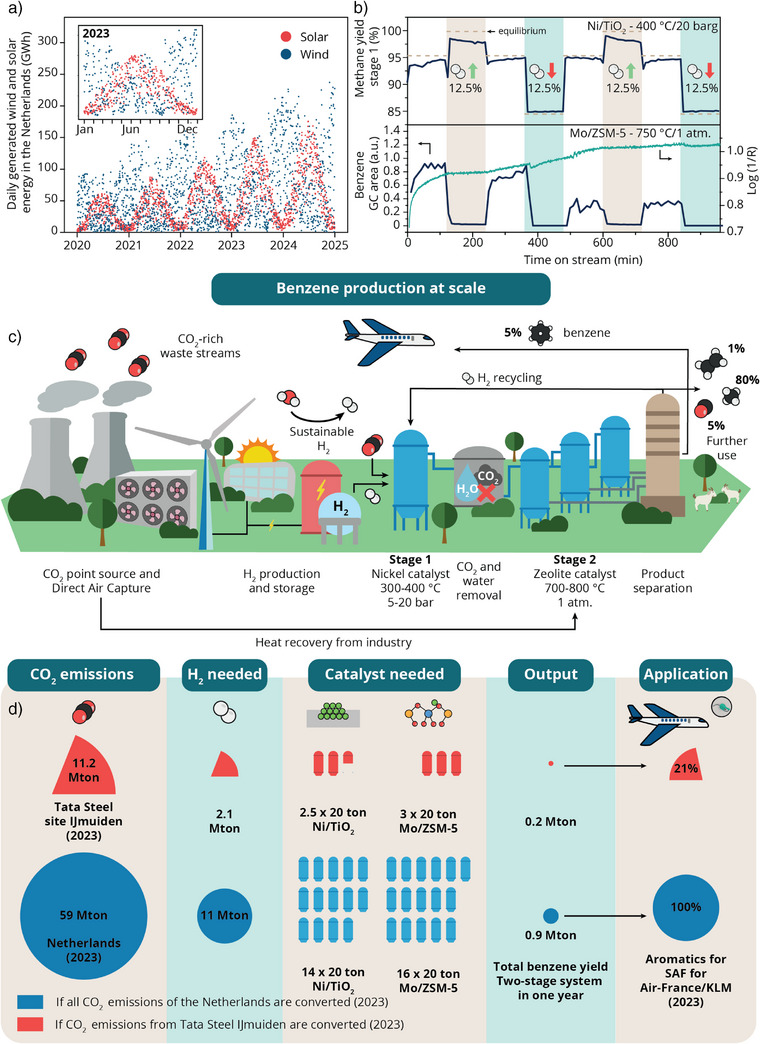
The implications of applying the two‐stage process at an industrial point source. a) Daily generated wind (blue) and solar (red) energy in the Netherlands, showing seasonal and daily fluctuations. Data from: Nationaal Energie Dashboard ned.nl. Figure adapted from ref. [57] b) The implications of a varying H_2_ supply on the output of the two‐stage process. Top panel: methane yield over time using a 15 wt.% Ni/TiO_2_ catalyst at 400 °C and 20 barg while H_2_ flow was alternated between stoichiometric (74.8 mL/min), 88.2 mL/min (H_2_ up, brown panel), and 65.5 mL/min (H_2_ down, green panel). Bottom panel: subsequent benzene yield (blue) over a 5 wt.% Mo/ZSM‐5 catalyst at 1 atm and 750 °C, and log(1/R) over time of the UV–vis absorption band at 17 230 cm^−1^ (green). c) Schematic representation of the application of the two‐stage process at scale near a point source. The CO_2_ from the point source is reacted with H_2_, obtained from water splitting using renewable energy, such as wind or solar. Following CO_2_ and water removal, the produced methane and residual H_2_ are fed to a second reactor for aromatization. Ideally, this is an integrated process, where heat from the metallurgical plant can be used to provide the energy for the energy‐intensive processes at stage 2. Some foreseen challenges in realizing the process are discussed in the text. d) The scale of benzene production based on the CO_2_ emissions of the Netherlands^[^ ^58^ ^]^ (blue) or the Tata Steel IJmuiden site^[^ ^59^ ^]^ (red). The amount of H_2_ as well as the amount of Ni/TiO_2_ and Mo/ZSM‐5 catalyst are shown to convert these CO_2_ emissions based on the current efficiencies of the lab‐scale process. Given this scale‐up is possible, this process could provide 21% or 100% of the aromatics for the SAF of KLM‐Air France using all the CO_2_ emissions from Tata Steel IJmuiden or the Netherlands respectively.

The question that remains is what the implication would be if the two‐stage process described in this work were to be implemented on a large scale. First of all, H_2_ is required for the methanation step, and to produce this sustainably through electrolysis, wind and solar energy are essential. However, wind currents and sun power vary naturally, resulting in fluctuations in the energy generated by wind turbines and solar panels. In Figure 4a, the energy generated by solar and wind (offshore and onshore) in the Netherlands between 2020 and 2025 is shown. It is immediately evident that there are strong fluctuations that would impact the production of H_2_. The catalyst system should be resilient toward the intermittency of the energy, and therefore for the intermittency of provided H_2_.

To test for resilience toward intermittency, we subjected our two‐stage process to fluctuating H_2_ flows. After an initial period of flowing CO_2_:H_2_ in a stoichiometric 1:4 ratio, we performed multiple steps where we either increased or decreased the H_2_ flow by 12.5% while keeping the CO_2_ and N_2_ flow constant. In the top panel of Figure 4b, the methane yields over the 15 wt.% Ni/TiO_2_ catalyst at 400 °C and 20 barg in stage 1 are shown together with the thermodynamic equilibrium values. With increasing H_2_ flows, higher CH_4_ yields are obtained, whereas decreasing H_2_ flows result in lower CH_4_ yields. In all cases, the system is operating at thermodynamic equilibrium. The Ni/TiO_2_ catalyst shows high stability, as for two cycles of H_2_ fluctuations we observe the same CH_4_ yields for the same ratios.

After two cycles of H_2_ fluctuations, we switched the exhaust of stage 1 to flow over stage 2 and performed the same H_2_ variations to study their effect on benzene production. Although the Ni/TiO_2_ catalyst was resilient toward H_2_ fluctuations, the benzene production completely dropped to zero for both an increase or decrease of 12.5% H_2_ in the initial feed. The H_2_ fluctuation translates to CO_2_:H_2_ ratios of 1:3.5 and 1:4.5, and thermodynamic calculations show that under these conditions, indeed, benzene formation is limited (Figure S8B). This supports our experimental observation that these very small fluctuations in gas feed completely suppress the benzene production. This highlights the sensitivity of the reaction system. Interestingly, after the Mo/ZSM‐5 catalyst was exposed to an abundance of H_2_ and returned to the initial CO_2_ to H_2_ ratio of 1:4, the benzene production recovered to its initial level. However, after a substoichiometric amount of H_2_ was present in the initial feed, the benzene production did not recover when we switched to CO_2_:H_2_ in a 1:4 ratio. Similar behavior has been observed earlier by Liu et al. in MDA co‐feeding experiments.^[^ ^34^ ^]^ After cofeeding H_2_ over a Mo/ZSM‐5 catalyst at 677 °C, the benzene production recovered when switching back to a CH_4_/Ar mixture, whereas this was not the case after cofeeding CO_2_.^[^ ^34^ ^]^ This showcases that the second stage of our process is highly sensitive to concentration variations. The concentration swings in this experiment were not as dramatic as the intermittency of renewable electricity. This emphasizes the need for H_2_ buffering in an industrial application to ensure continuous benzene production.

During the H_2_ fluctuation experiment, operando UV–vis diffuse reflectance spectroscopy was performed to monitor coke deposition on the Mo/ZSM‐5 catalyst surface. In the bottom panel of Figure 4b, the log(1/R) value for the UV–vis absorption band at 17 230 cm^−1^ is shown with time on stream. Over the entire reaction, the band grew in intensity, but a slight decrease was observed with decreasing H_2_ content (i.e., a relatively higher amount of residual CO_2_ from stage 1). This indicates that increased CO_2_ levels are capable of removing coke from the catalyst surface by reacting with the coke species on the surface, while the increased H_2_ levels were not sufficient to actively remove the formed coke deposition. However, to retrieve complete catalytic activation, it would be necessary to activate the Mo/ZSM‐5 catalyst (e.g., in H_2_ or CH_4_).

To understand how the potential of our system as an alternative route to produce aromatics, we calculated the possible scale of operation based on current CO_2_ emissions in the Netherlands (see Supporting Information for details, Figure 4c,d). The metallurgical industry is known to emit large amounts of CO_2_. In the Netherlands, the site of Tata Steel IJmuiden is the single largest CO_2_ emitter. Of the ∼ 60 Mton CO_2_ emitted in the Netherlands in 2023,^[^ ^58^ ^]^ the Tata Steel IJmuiden site was responsible for ∼ 11.2 Mton.^[^ ^59^ ^]^ About half of these emissions come from the steel manufacturing processing itself, and half of the emissions come from the production of electricity (by Vattenfall in Velsen‐North). Assuming all the CO_2_ from this site can be captured and converted to benzene with 5% efficiency, 0.2 Mton benzene can be produced from this site on an annual basis. Looking back at the example of the aromatics required for producing jet fuel, the amount of benzene produced from CO_2_ at the IJmuiden site would have been enough to provide ∼ 21% of the total fuel consumption of the Air‐France/KLM fleet in 2023.^[^ ^60^ ^]^ All the CO_2_ emissions in the Netherlands would be required to produce 100% of the aromatics required for jet fuel consumption. With the current efficiency and catalyst use of the lab‐scale process, this translates to ∼ 2.5 20‐ton reactors of Ni/TiO_2_ catalyst and 3 20‐ton reactors of Mo/ZSM‐5 zeolite catalyst at the IJmuiden site (or respectively 14 and 16 20‐ton reactors to convert all CO_2_ emissions of the Netherlands). The CO_2_ methanation reaction is already applied at larger scales,^[^ ^41^, ^61^ ^]^ but a full scale‐up of the MDA reaction is not yet operational. Efforts are being made to bring this reaction to a bigger scale, and when scaling up, the catalytic performance could significantly change (for better or worse).^[^ ^62^, ^63^, ^64^ ^]^

To successfully scale up this process, a techno‐economical assessment should be performed, and many challenges are still to be considered. Based on our back‐of‐the‐envelope calculations, the process requires H_2_ in the order of magnitude of megatons. Ideally, the H_2_ is produced sustainably through electrolysis using wind and solar energy. Assuming 50–58 kWh of power is required for the production of 1 kg H_2_, we calculated the amount of renewable energy required to produce the amount of H_2_ to convert all the IJmuiden CO_2_ emissions from the Netherlands or the Tata Steel site on a daily basis. This amounts to ∼ 1600 GWh and ∼ 310 GWh of sustainable energy to convert all the emissions of the Netherlands and Tata Steel IJmuiden, respectively. Comparing these numbers to the total amount of sustainable energy produced in the Netherlands (Figure 4a), it becomes evident that is essential to recycle the H_2_ released after the second reaction and use it again for the CO_2_ methanation reaction.

As shown before, fluctuations in H_2_ and CO_2_ could be detrimental to the zeolite catalyst, showing that H_2_ or electric energy storage is a necessity to be able to meet sudden changes in supply and demand. Furthermore, H_2_O removal is essential; in our lab scale reactor water removal was executed using a cold trap and molecular sieves, but ideally, other techniques such as membranes are utilized to limit the loss of energy between reactor stages. To ensure continuous benzene production, one should consider implementing multiple MDA reactors to compensate for catalyst deactivation over time. For example, one could implement three reactors to carry out the reaction, catalyst activation, and regeneration in parallel.

To fully close the carbon cycle of this process, direct air capture (DAC) units could be placed on‐site. Furthermore, technical strategies can be used to recover industrial waste heat to provide the energy for the high‐temperature process (i.e., stage 2, MDA). Product separation also needs to be taken into account. Ideally, the formed H_2_ from stage 2 is recycled and reused in reactor 1 to limit the use of H_2_ produced via electrolysis. Separation of CH_4_ and H_2_ can be challenging and energy intensive, yet membrane technologies are being developed for efficient gas separation.^[^ ^65^, ^66^ ^]^ The other products formed in the two‐stage process (i.e., CO and ethylene) can be used as input for other processes such as FTS or polymerization resulting in hydrocarbons and plastics, respectively. Furthermore, in this scenario, ∼ 80% of methane exits stage 2 unreacted. Ideally, this methane is used efficiently by either (1) recycling it for further aromatization, (2) pyrolyzing it to provide additional H_2_ and solid carbon which can be utilized as carbon source for steel making, or (3) be directly injected into the natural gas grid for further use.

## Conclusion

Combining two thermocatalytic reactions, namely CO_2_ methanation over Ni/TiO_2_ and methane dehydroaromatization over Mo/ZSM‐5, resulted in the selective production of benzene from CO_2_ and H_2_. Guided by thermodynamic calculations, we explored the boundary conditions of the two‐stage process: to produce aromatics, the CH_4_ yields in the first reactor must be at least 93%, the initial feed must have a stoichiometric 1:4 CO_2_:H_2_ ratio, and H_2_O must be removed after the first reaction. The remaining CO_2_ and H_2_ in the CH_4_ feed to the second reactor are advantageous, as they stabilize the benzene production over time. As a result, the methane dehydroaromatization reaction could be run for at least 72 h. Using operando UV–vis diffuse reflectance spectroscopy, we found that the CO_2_ and H_2_ inhibit the carbon growth on the Mo/ZSM‐5 catalyst, which is the usual cause of deactivation in the methane dehydroaromatization reaction. This system could potentially be used in industry to shift from petroleum‐based benzene–toluene–xylene (BTX) to C1‐based BTX. Several challenges remain, such as designing the system in a way that industrial waste heat, plenty available from, e.g., a metallurgical blast furnace, is used to heat the two reactors. Also, H_2_ separation from unreacted methane should be implemented to recycle and reuse the H_2_ formed in the second reactor in the CO_2_ methanation reaction. On top of that, the intermittency of H_2_ produced from sustainable sources due to weather fluctuations is detrimental to the benzene production. One should implement a H_2_ storage unit on‐site to accommodate for the fluctuations in H_2_ production. Overall, we have showcased a feasible route for the production of aromatics, which could be applied in various industries where CO_2_ and/or CH_4_ are released in high concentrations, such as the metallurgical and cement industries.

## Supporting Information

The authors have cited additional references within the Supporting Information which can be found in that document.

## Conflict of Interests

The authors declare no conflict of interest.

## Supporting information



Supporting information

## Data Availability

The data that support the findings of this study are available from the corresponding author upon reasonable request.
