# Supplementary material for: Two‐Stage Catalytic Conversion of Carbon Dioxide Into Aromatics Via Methane
Source: Angew Chem Int Ed Engl. 2025 Oct 21;64(50):e202517563. doi: 10.1002/anie.202517563 (PMC12684318; doi:10.1002/anie.202517563)
Supplement: Supplementary file 1 — Supporting information [file ANIE-64-e202517563-s001.docx]

Supporting Information

**Two-Stage Catalytic Conversion of Carbon Dioxide into Aromatics via Methane**

J.J.G. Kromwijk^[a],[b]^, A.E.M. Melcherts^[a],[b]^, L. de Jong^[a]^, J.F. van Leusden^[a]^, J.C.L. Janssens^[a]^, R. Oord^[a]^, W. van der Stam^[a]^, M. Monai^[a]^, and B.M. Weckhuysen*^[a]^

[a] Inorganic Chemistry and Catalysis group, Institute for Sustainable and Circular Chemistry, Utrecht University, Universiteitsweg 99, 3584 CG Utrecht (the Netherlands)
* E-mail: [b.m.weckhuysen@uu.nl](mailto:b.m.weckhuysen@uu.nl)

[b] These authors contributed equally: J.J.G. Kromwijk and A.E.M. Melcherts

Table of Contents

[1. Experimental Methods 3](#_Toc208909738)

[1.1 Thermodynamic calculations 3](#_Toc208909739)

[1.2 Catalyst preparation 3](#_Toc208909740)

[1.3 Catalyst Characterization 4](#_Toc208909741)

[1.4 Catalyst testing 4](#_Toc208909742)

[1.4.1 CO_2_ methanation 4](#_Toc208909743)

[1.4.2 Methane dehydroaromatization 5](#_Toc208909744)

[1.4.3 Two-stage conversion of CO_2_ into benzene 6](#_Toc208909745)

[1.4.4 Two-stage conversion of CO_2_ into benzene with H_2_ flow fluctuations 7](#_Toc208909746)

[2. Two-Stage Reactor Design 8](#_Toc208909747)

[3. Choice of catalyst materials 10](#_Toc208909748)

[3.1 Ni/TiO_2_ for CO_2_ methanation 10](#_Toc208909749)

[3.2 Mo/ZSM-5 for methane dehydroaromatization 10](#_Toc208909750)

[4. Catalyst characterization 12](#_Toc208909751)

[5. Two-Stage Conversion of CO_2_ into Aromatics 13](#_Toc208909752)

[4.1 Catalytic performance 13](#_Toc208909753)

[4.2 Operando UV-Vis Diffuse Reflectance Spectroscopy 13](#_Toc208909754)

[4.3 Used catalyst materials characterization 14](#_Toc208909755)

[6. Thermodynamic calculations 15](#_Toc208909756)

[7. Two-Stage Simulation Experiments 16](#_Toc208909757)

[6.1 Catalytic performance in the MDA reaction 16](#_Toc208909758)

[6.2 Operando UV-Vis diffuse reflectance spectroscopy 17](#_Toc208909759)

[6.3 Used catalysts materials characterization 18](#_Toc208909760)

[8. Back-of-the-envelope calculations for scale of the process 19](#_Toc208909761)

[9. References 20](#_Toc208909762)

# Experimental Methods

## 1.1 Thermodynamic calculations

Equilibrium calculations were performed using the HSC Chemistry 9.1 software in the Gem equilibrium composition module by the Gibbs free energy minimization method.

The thermodynamic benzene yield as a function of temperature at 1 bar, shown in **Figure S8D**, was calculated using 19.6 kmol CH_4_ and 2 kmol N_2_ as input. Benzene, CO_2_, CO, H_2_, and H_2_O were included as possible components. Solid carbon was added as input to calculate the benzene yield assuming coke formation.

The thermodynamic CH_4_ yield as a function of temperature, at 1 bar, 6 bar, 11 bar, and 21 bar, shown in **Figure 2E–H** was calculated using 19.6 kmol CO_2_, 78.4 kmol H_2_, and 2 kmol N_2_ as input. CO, CH_4_, and H_2_O were included as possible components.

To study the effect of CH_4_ yield from stage 1 on the benzene production in stage 2, thermodynamic calculations were performed at 700, 750, and 800 °C, and 1 bar. The input for these calculations is shown in **Table S1**. As a starting point, we assumed 100 % CO_2_ conversion in stage 1 using three different CO_2_:H_2_ ratios (1:3.5, 1:4, and 1:6) as input. Per step, an x amount of reagent and product were respectively added or removed in stoichiometric amounts, with one step representing a decrease in CH_4_ yield of 1 %. Assuming all the H_2_O can be removed between the two reactions, we did not use this component in our calculation. This component was only added for the calculation shown in **Figure S8A**, to showcase the effect of H_2_O on the benzene production. To show the effect of CO_2_ removal on the benzene production in **Figure S8C**, the ‘Add x amount’ was set to zero.

**Table S1** Input for the thermodynamic calculations in the HSC Chemistry 9.1 Gem equilibrium mode to study the effect of varying CH_4_ yields from stage 1 on the benzene production in stage 2.

| **CO_2_:H_2_ ratio** | **1:3.2** | | **1:4** | | **1:6** | |
| --- | --- | --- | --- | --- | --- | --- |
| **Components** | **Start amount (kmol)** | **Add x amount (kmol)** | **Start amount (kmol)** | **Add x amount (kmol)** | **Start amount (kmol)** | **Add x amount (kmol)** |
| CO_2_ | 3.92 | +0.196 | 0 | +0.196** | 0 | +0.196 |
| H_2_ | 0 | +0.784 | 0 | +0.784 | 39.2 | +0.784 |
| N_2_ | 2 | 0 | 2 | 0 | 2 | 0 |
| CH_4_ | 15.68 | -0.196 | 19.6 | -0.196 | 19.6 | -0.196 |
| CO | 0 | 0 | 0 | 0 | 0 | 0 |
| C_6_H_6_ | 0 | 0 | 0 | 0 | 0 | 0 |
| H_2_O* | 0 | 0 | 39.2 | -0.392 | 0 | 0 |

* H_2_O is only included in the thermodynamic calculation shown in **Figure S8A** to show the effect of H_2_O on benzene production
** Number set to zero in the thermodynamic calculation shown in **Figure S8C** to show the effect of CO_2_ removal

## 1.2 Catalyst preparation

**Ni/TiO_2_ (15 wt.%)** 8.76 g of Ni nitrate hexahydrate (Ni(NO_3_)_3_.6H_2_O, Sigma Aldrich, EMSURE ACS for analysis) and 11.0 g of urea (Acros Organics, 99.5 %, for analysis) were dissolved in 1.2 L demi H_2_O in a double walled vessel. 10.0 g of TiO_2_ P25 (Acros Organics, with a BET surface area of 42 m^2^/g and pore volume of 0.18 cm^3^/g) was added. The mixture was heavily stirred with a mechanical stirrer and heated to 90 °C for 20 h. The suspension was washed by centrifugation until the pH of the supernatant was neutral. The catalyst was dried in an 80 °C static oven overnight.

**Mo/ZSM-5 (5 wt.%)** Zeolite H-ZSM-5 was obtained by calcining NH_4_-ZSM-5 (CBV 2314, Zeolyst) in static air at 550 °C for 7 h using a heating ramp of 2 °C/min. Before incipient wetness impregnation, H-ZSM-5 was dried for 4 h at 120 °C under vacuum in a round-bottom flask. A 2.6 M molybdenum stock solution was prepared by dissolving the appropriate amount of ammonium heptamolybdate tetrahydrate (VWR Chemicals) in ultrapure water in a volumetric flask. Using a syringe, the amount of solution required to fill the pores of the zeolite (0.21 mL/g) was measured off and slowly dropped to the zeolite under vacuum. After mixing, a light-yellow powder was obtained which was dried overnight at 80 °C and calcined in static air at 550 °C for 7 h using a 2 °C/min heating ramp resulting in a 5 wt.% Mo/ZSM-5 catalyst.

## 1.3 Catalyst Characterization

**ICP-OES** Elemental analysis was carried out at Mikroanalytisches Laboratorium Kolbe, Germany, with an inductively coupled plasma (ICP) optical emission spectroscopy (OES) instrument (PerkinElmer, Waltham, MA, USA) after sample dissolution according to their standard in-house procedures.

**Thermogravimetric Analysis** To analyze the coke content of the spent catalyst materials, the samples were analyzed using thermogravimetric analysis (TGA) performed on a Perkin Elmer TGA 8000 instrument. In a typical experiment, 20–30 mg of spent catalyst was loaded in a crucible and heated from 30 to 150 °C with a 10 °C/min ramp and held for 60 min at this temperature to dry the sample under a 45.0 mL/min flow of air. Consequently, the sample was heated to 800 °C. The measured weight at this temperature was compared to the initial weight to determine the coke content on the sample.

**Temperature-Programmed Reduction** Temperature-Programmed Reduction (TPR) profiles were obtained using an Altamira Instruments AMI-300IP. Approximately 25 mg of the Ni/TiO_2_ catalyst was dried at 120 °C for 15 min (10°C/min) under Ar. At 40 °C (5°C/min) a mixture of 5% H_2_/Ar (total flow 25 mL/min) was introduced for 15 minutes, before ramping up to 900 °C (5°C/min) under 5% H_2_/Ar.

**HAADF-STEM** High-angle annular dark-field scanning transmission electron microscopy (HAADF-STEM) images and energy-dispersive X-ray (X-ray) elemental maps were recorded on the Spectra 300 (Thermo Fisher Scientific) operating at 300 kV. STEM samples were prepared by depositing crushed catalyst on 300 mesh Cu grids with a Formvar/Carbon film.

## 1.4 Catalyst testing

### 1.4.1 CO_2_ methanation

To test the performance of the CO_2_ methanation process, 500 mg of 15 wt.% Ni/TiO_2_ (125–212 μm) was loaded in a SiO_2_-coated stainless steel reaction tube with an inner diameter of 6.95 mm. The reactor was placed in a custom-built oven (oven 1) and heated to 400 °C with a 5 °C/min ramp under a 2:1:1 H_2_/N_2_/He flow (total flow 80 mL/min). After 1 h reduction, the gasses were switched at 400 °C to 19.6 mL/min CO_2_, 78.4 mL/min H_2_, and 2 mL/min N_2_. Subsequently, the pressure was built up to 20 barg at a rate of 1 bar/min using a HT Series Equilbar Precision Back Pressure Regulator, kept at 130 °C in a muffle oven (intermediate oven). The temperature was kept for 90 min. This step was followed by 90 min isotherms at 380, 360, 340, 320 °C and subsequently at 340, 360, 380, and 400 °C. The full temperature cycle was repeated at 10, 5, and 0 barg.

Online activity and selectivity measurements were performed with an Interscience Trace 1300 gas chromatograph (GC) equipped with an FID channel for the detection of C1–C5 hydrocarbons, an FID channel for the detection of alcohols and hydrocarbons, and a TCD channel for the detection of light gasses (CO_2_, CH_4_, N_2_, and CO).

The total flow into the GC was calculated based on:

$$Total flow, out= \frac{Total Flow, in}{{1+4*\%}_{{CH}_{4},out} {+ \%}_{CO,out}}$$

where ${\%}_{i,out}$ ($i$= CH_4_ or CO) is the average integrated peak converted to % based on GC calibrations for multiple injections at each temperature and pressure interval.

Carbon yields were calculated according to:

$$Y_{i}=\frac{F_{i,out}}{F_{CO_{2},in}} \times x\times100\%$$

where $F_{i}$ denotes the molar flow of molecule $i$.

The selectivity was calculated according to:

$$S_{i}=\frac{Y_{i}}{Y_{CH_{4}}+Y_{CO}} \times x\times100\%$$

### 1.4.2 Methane dehydroaromatization

In a typical run, 600 mg of sample (212–425 μm) was loaded in a quartz fixed bed reactor with an internal diameter of 6.95 mm and was placed in a Carbolite Gero TG3 tubular furnace, with three separate temperature zones. In all experiments, the three temperature zones were kept at the same level. The sample was heated with a 5 °C/min ramp to 150 °C in a 20 mL/min N_2_ flow controlled by Bronkhorst Prestige mass flow controllers. At 150 °C, the gasses were switched to the reaction mixture (**Table S2**) and held for 2 h as a reference point for the conversion calculations. After this, the reactor was heated to the reaction temperature (700, 750, or 800 °C) with a 5 °C/min ramp under 20 mL/min N_2_. When the reaction temperature was achieved, the gases were switched to the reaction mixture (**Table S2**) for 10 h unless otherwise mentioned.

**Table S2** Reaction mixtures used for the methane dehydroaromatization (MDA) experiments to simulate the two-stage conversion of CO_2_ to benzene considering various CH_4_ yields in the first reactor. To calculate these flows, a 1:4 CO_2_:H_2_ ratio in the first reactor was considered as well as complete H_2_O removal between the reactors and 100 % selectivity towards CH_4_.

|  | **Reaction mixture** | | | | |
| --- | --- | --- | --- | --- | --- |
| **Condition** | **CH_4_ (mL/min)** | **CO_2_ (mL/min)** | **H_2_ (mL/min)** | **N_2_ (mL/min)** | **Total flow (mL/min)** |
| 100 % CH_4_ yield | 19.6 | - | - | 2 | 21.6 |
| 95 % CH_4_ yield | 18.62 | 0.98 | 3.92 | 2 | 25.52 |
| 93 % CH_4_ yield | 18.23 | 1.37 | 5.49 | 2 | 27.09 |

Online activity and selectivity measurements were performed with an Interscience Trace 1300 gas chromatograph (GC) equipped with an FID channel for the detection of C_1_–C_5_ hydrocarbons, an FID channel for the detection of alcohols and hydrocarbons, and a TCD channel for the detection of light gasses (CO_2_, CH_4_, N_2_, and CO).

Conversions were calculated according to:

$$X_{reactant}= \frac{\left( \frac{A_{{CH}_{4},in}}{A_{N_{2},in}} \right)-\left( \frac{A_{{CH}_{4},out}}{A_{N_{2},out}} \right)}{\frac{A_{{CH}_{4},in}}{A_{N_{2},in}}} \times100\%$$

where $A_{i,in}$ ($i$= CH_4_ or N_2_) is the average integrated peak area of three injections recorded at 150 °C before the reaction. Carbon yields were calculated according to:

$$Y_{product}=\frac{F_{C_{x}H_{y},out}}{F_{CH_{4}}} \times x\times100\%$$

where $F_{i}$ denotes the molar flow of molecule $i$ , and $x$ and $y$ denote the number of carbon and hydrogen atoms in a product molecule. In case CO_2_ was part of the reaction mixture, the CO yield was calculated according to:

$$Y_{product}=\frac{F_{C_{x}H_{y},out}}{F_{CH_{4}}+F_{{CO}_{2}}} \times x\times100\%$$

UV-Vis diffuse reflectance spectroscopy (DRS) was performed during the reaction using an AcaSpec2048L spectrometer equipped with a 100 μm slit connected to a high-temperature UV-Vis optical fiber probe. The spectra were collected every 30 s in reflection mode. A dark spectrum was recorded at room temperature and $log(1/R)$ was calculated using:

$$\log(1/R)={log}_{10} (\frac{I_{0}}{I})$$

where $I$ is the intensity spectrum recorded during the reaction and $I_{0}$ is the first intensity spectrum recorded at reaction temperature.

### 1.4.3 Two-stage conversion of CO_2_ into benzene

For the two-stage reaction, 500 mg of 15 wt.% Ni/TiO_2_ (125–212 μm) was loaded in a SiO_2_-coated stainless steel reaction tube with an inner diameter of 6.95 mm. The reactor was placed in a custom-built oven (oven 1) and heated to 400 °C with a 5 °C/min ramp under a 2:1:1 H_2_/N_2_/He flow (total flow 80 mL/min). After 1 h of reduction, the gasses were switched at 400 °C to 19.6 mL/min CO_2_, 78.4 mL/min H_2_, and 2 mL/min N_2_. Subsequently, the pressure was built up to 20 barg at a 1 bar/min rate using a HT Series Equilbar Precision Back Pressure Regulator, kept at 180 °C in a muffle oven (intermediate oven) to prevent water condensation while also maintaining a suitable temperature for the O-rings in the connectors. The reaction was held for 20 h to obtain stable CO_2_ conversion levels. The outlet flow was passed through a condenser with ethylene glycol cooled at 5 °C and a quartz tube (ID = 6.95 mm) containing 3–5 g of molecular sieves, 3Å (Sigma Aldrich, crushed beads 4–8 mesh, pre-dried under N_2_ flow at 300 °C for 10 h) to remove the formed water, before flowing it to the second reactor oven.

A quartz tube (ID = 6.95 mm) with 600 mg 5 wt.% Mo/ZSM-5 (212–425 μm) was heated to reaction temperature with a 5 °C/min ramp in a Carbolite Gero TG3 tubular oven (oven 2) under a 20 mL/min N_2_ flow. It was ensured the reactor was at the reaction temperature after the CO_2_ methanation was performed for 20 h. When the reaction temperature was reached, the N_2_ flow was turned off and the valves in the intermediate oven were switched so that the outlet flow of oven 1, and thus the products of the CO_2_ methanation reaction, were directed towards oven 2. During the two-stage reaction, UV-Vis diffuse reflectance spectroscopy was performed on the Mo/ZSM-5 catalyst as described in the section above.

### 1.4.4 Two-stage conversion of CO_2_ into benzene with H_2_ flow fluctuations

To simulate fluctuation in H_2_ production, the two-stage conversion of CO_2_ to benzene was performed with fluctuating H_2_ flows. CO_2_ hydrogenation was monitored over a 15 wt.% Ni/TiO_2_ catalyst (500 mg, 125–212 µm, 400 °C, 20 barg, pre-reduced for 1 h in situ at 400 °C). Two consecutive cycles were executed of (1) 2 h CO_2_:H_2_ =1:4 flow, (2) 2 h CO_2_:H_2_ =1:4.5 flow, (3) 2 h CO_2_:H_2_ =1:4 flow, (4) 2 h CO_2_:H_2_ =1:3.5 flow. The two cycles concluded with 4 h of stoichiometric flow to stabilize the CO_2_ conversion before switching the gas feed from stage 1 to stage 2. Here a 5 wt.% Mo/ZSM-5 catalyst (600 mg, 212–425 µm) was heated to 750 °C (5 °C/min in 20 mL/min N_2_ flow). After the switch the two consecutive cycles were repeated to monitor the effect on benzene production. **Table S3** shows the corresponding flows of the different steps.

**Table S3** Reaction mixtures used for the two-stage conversion of CO_2_ to benzene experiments with fluctuating H_2_ flows.

|  | **Reaction mixture** | | | | |
| --- | --- | --- | --- | --- | --- |
| **Condition** | **CO_2_:H_2_ ratio** | **CO_2_ (mL/min)** | **H_2_ (mL/min)** | **N_2_ (mL/min)** | **Total flow (mL/min)** |
| Stoichiometric | 1:4 | 19.6 | 78.4 | 2 | 100 |
| 12.5 % higher H_2_ | 1:4.5 | 19.6 | 88.2 | 2 | 109.8 |
| 12.5 % lower H_2_ | 1:3.5 | 19.6 | 68.6 | 2 | 90.2 |

# Two-Stage Reactor Design


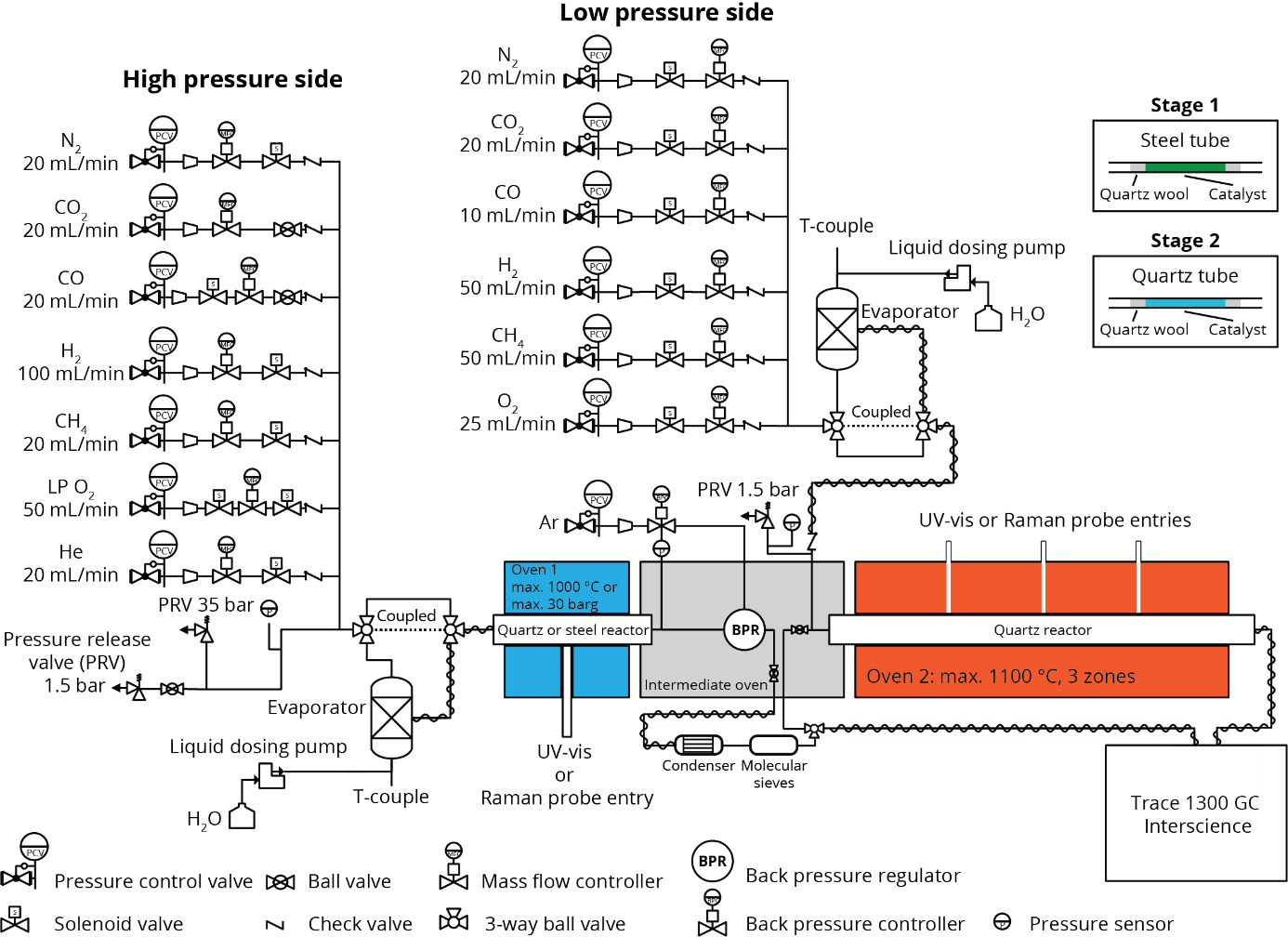
**Figure S1** Technical drawing of the two-stage reactor set-up for the conversion of CO_2_ into benzene via CH_4_.


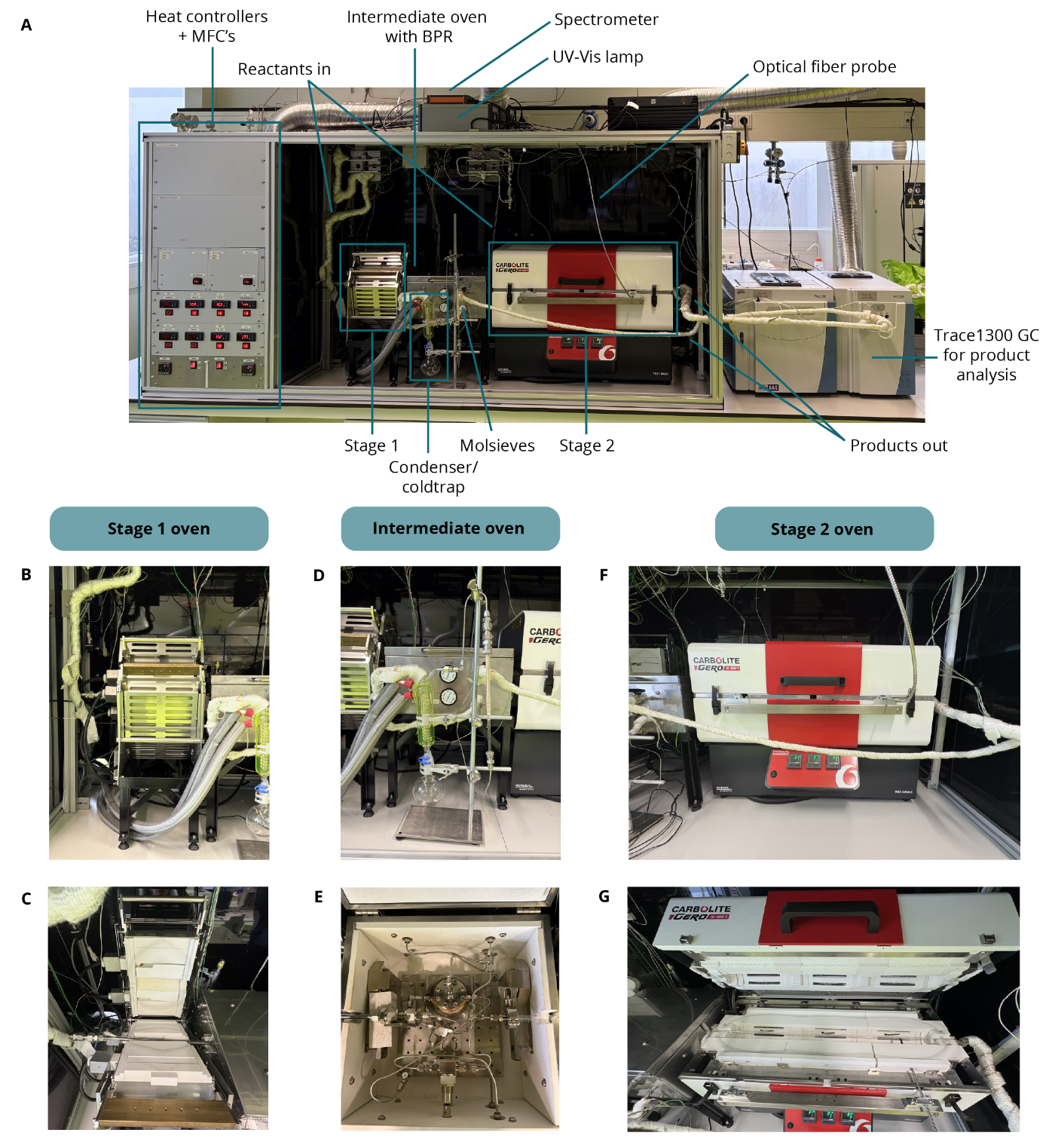


**Figure S2** Pictures of two-stage reactor set-up for the conversion of CO_2_ into benzene via CH_4_ used in this work. (**A**) A complete overview of the reactor set-up including the heat controllers and MFCs, the Trace 1300 gas chromatograph used for product analysis, (**B,C**) the oven for stage 1, where a steel reactor can be installed with vacuum coupling O-ring (VCO) fittings and Kalrez connectors (**D,E**) the intermediate oven with back-pressure regulator (BPR), the ethylene glycol condenser, and the reactor with molecular sieves, (**F,G**) the oven for stage 2, with three zones which can be operated at different temperatures, and holes for optical fiber probes for measuring operando UV-Vis spectroscopy. In this oven, a quartz reactor can be installed using Kalrez and Klein Flansch (KF) Swagelok adapter connectors.

# Choice of catalyst materials

## Ni/TiO_2_ for CO_2_ methanation

Noble metals supported on metal oxides were found among the best performing catalysts for the CO_2_ methanation reaction, yet their relative high costs make the use of non-noble metals such as Ni and Co more applicable. Among these metals, Ni is widely studied due to its relatively high methanation activity and selectivity.^[1]^ The choice of metal oxide support can further influence the performance. Among the tested supported Ni catalysts by Vogt et al. titania-supported Ni catalysts showed a high activity and selectivity towards CH_4_ compared to other supports, like SiO_2_ or Al_2_O_3_.^[2,3]^ The high selectivity towards methane was favorable in this study to mitigate the effects of CO or other impurities. Moreover, the use of reducible oxides, such as TiO_2_, offers great flexibility in performance due to the tunability in strong metal-support interactions affecting both structural and electronic properties and its ability to open up different catalytic pathways by facilitating different reaction intermediates on the support or metal-support interface.^[4]^

## Mo/ZSM-5 for methane dehydroaromatization

Metal-modified zeolite materials are typically used for the methane dehydroaromatization (MDA) reaction. Since 1993, when Wang et al. showed that incorporating Mo into zeolite ZSM-5 resulted in CH_4_ conversions of 7–8 % with high benzene selectivity, no other catalyst has been found that outperforms this metal-zeolite combination in the MDA reaction.^[5]^ Both the nature of the zeolite as well as the metal influence the catalytic performance.

The Brønsted acid sites (BAS) in the zeolite play several roles. Not only do they provide anchoring sites for the metal, but there is also strong evidence that the acid sites serve as active site for aromatization.^[6–8]^ Furthermore, the framework topology of the zeolite material plays an important role; zeolites that contain pore diameters of a similar size as the dynamic diameter of benzene (~ 6 Å), such as ZSM-5, ZSM-8, and ZSM-11, are excellent zeolite supports for MDA catalysts.^[9]^ Zeolites with smaller pores, like SAPO-34 have a higher selectivity towards smaller hydrocarbons, whereas MDA catalysts, for which zeolites with bigger pores are used, tend to deactivate faster due to excessive coke formation.^[9–11]^ In most MDA studies, zeolite ZSM-5 is used as support material.

To understand the role of molybdenum as an active site, operando studies have been performed. In a study where operando X-ray Absorption Spectroscopy (XAS) and X-ray Emission Spectroscopy (XES) were combined, it was shown that partially carburized molybdenum oxide species (MoC_x_O_y_) can activate CH_4_.^[12]^ Upon further carburization, Mo carbides are formed, which partially detach from the framework, but are necessary to form aromatic products.

# Catalyst characterization


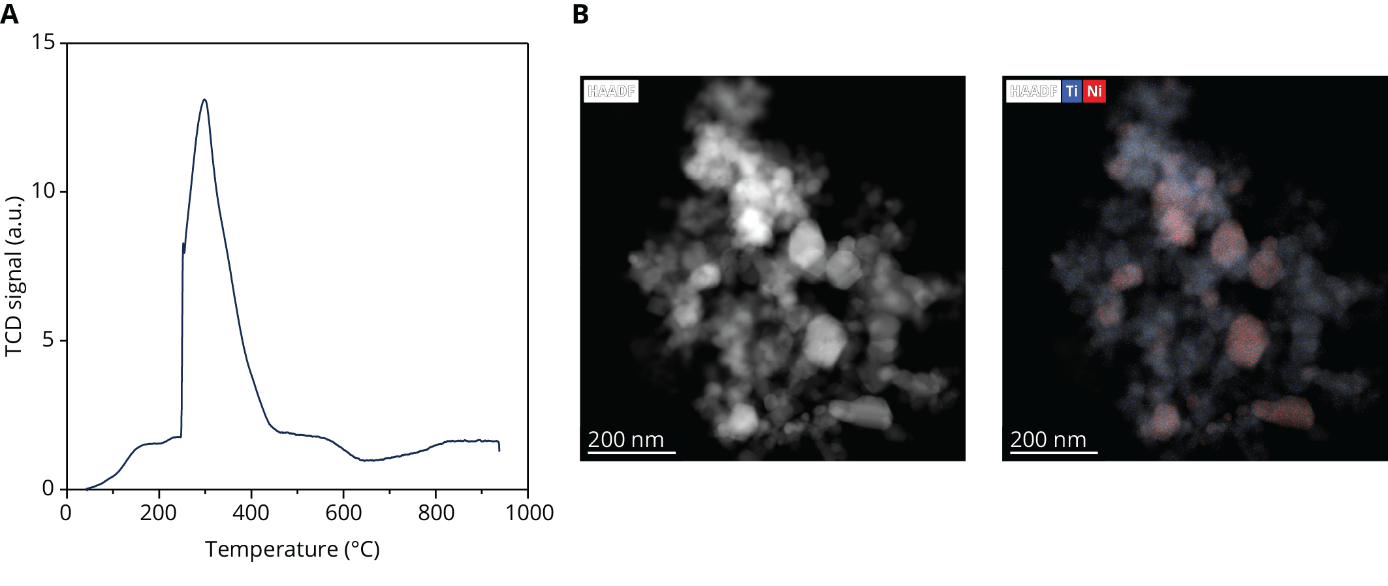


**Figure S3** (**A**)Temperature programmed reduction (TPR) of the synthesized 15 wt.% Ni/TiO_2_ catalyst. (**B**) High-angle annular dark-field scanning transmission electron microscopy (HAADF-STEM) images and corresponding Energy-dispersive X-ray spectroscopy (EDX) maps of the 15 wt.% Ni/TiO_2_ catalyst used after the two-stage experiment at 750 °C indicating the Ti (blue) and the Ni (red).

**Table S4** Metal content of the catalyst samples used in this work as determined with Inductively-Coupled Plasma Optical Emission Spectroscopy (ICP-OES).

| **Samples** | **Used in experiments** | **Metal content (wt.%)** |
| --- | --- | --- |
| 5 wt.% Mo/ZSM-5 | MDA simulations at 700 and 750 °C | 4.9* |
| 5 wt.% Mo/ZSM-5 | MDA simulations at 800 °C | 4.5* |
| 5 wt.% Mo/ZSM-5 | Two-stage reaction and intermittency | 4.3* |
| 15 wt.% Ni/TiO_2_ | All reported CO_2_ hydrogenation experiments and two-stage reactions | 14.4** |

* Molybdenum content
** Nickel content

# Two-Stage Conversion of CO_2_ into Aromatics

## 4.1 Catalytic performance


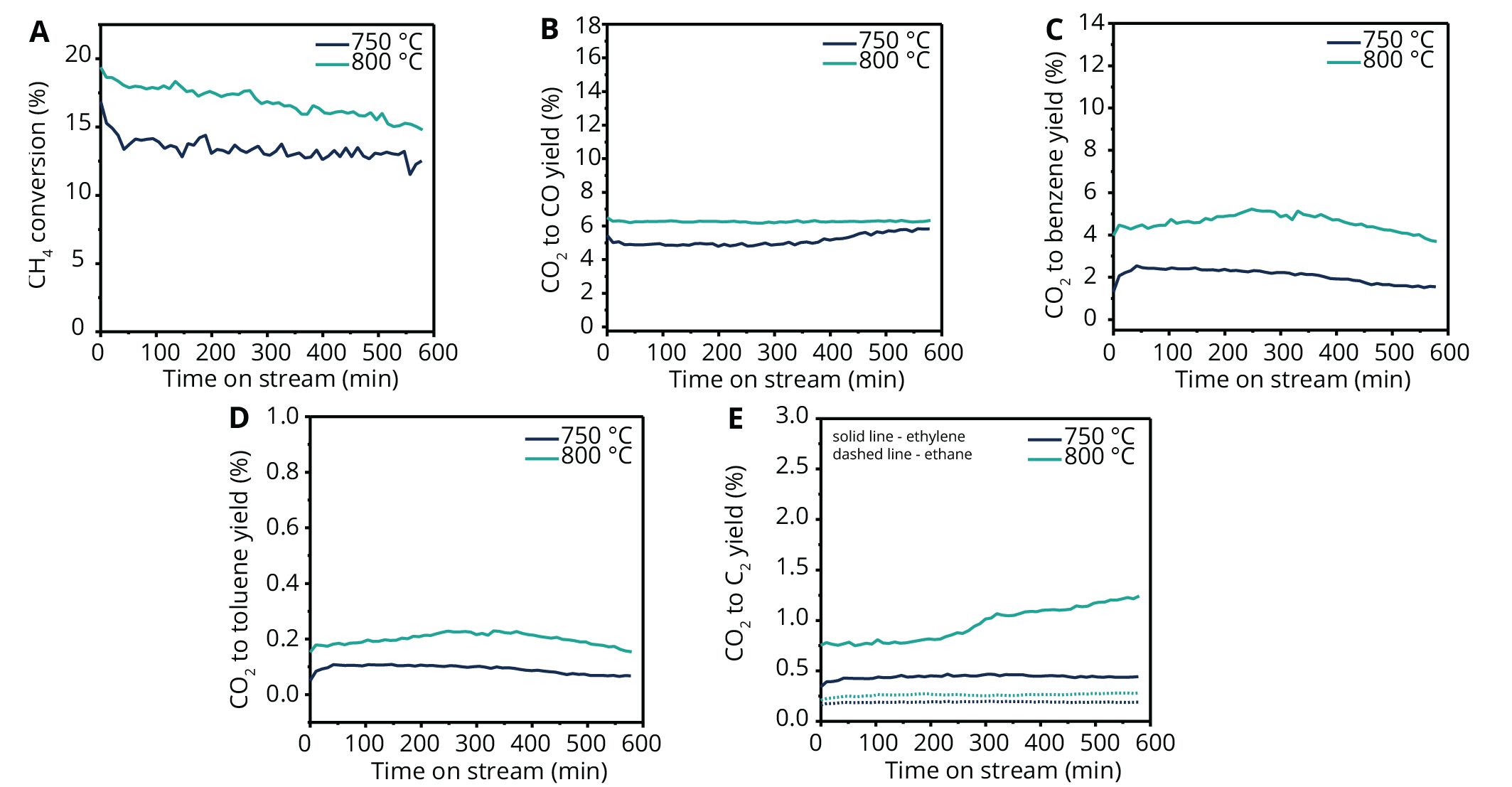


**Figure S4** Catalytic performance of the 5 wt.% Mo/ZSM-5 catalyst during the two-stage conversion of CO_2_ into aromatics at 750 (blue) and 800 °C (green) expressed as (**A**) CH_4_ conversion, (**B**) CO_2_ and CH_4_ to CO yield , (**C**) CO_2_ to benzene yield (**D**) CO_2_ to toluene yield (**E**) CO_2_ to ethane (solid) and ethylene (dashed) yield.

## 4.2 Operando UV-Vis Diffuse Reflectance Spectroscopy


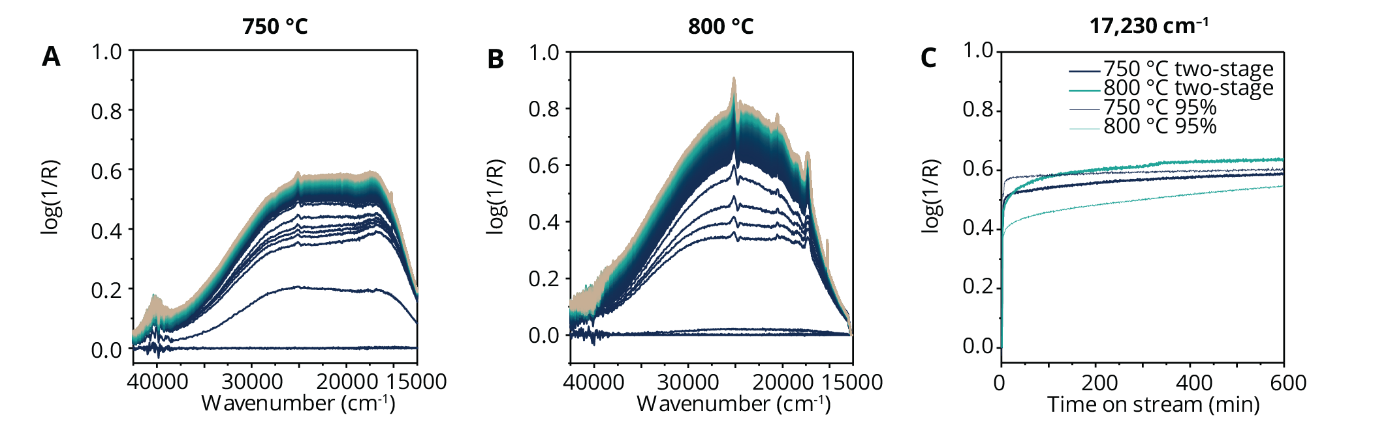


**Figure S5** Operando UV-Vis diffuse reflectance spectra recorded on the MDA reaction during the two-stage reaction (**A**) at 750 and (**B**) 800 °C. (**C**) The respective trends of log(1/R) over time at 17,230 cm^–1^ during the two-stage reaction (thick lines) and during simulation experiments where the gas composition was resembling the 95 % CH_4_ yield (CH_4_:CO_2_:H_2_:N_2_ = 18.62:0.98:3.92:2, thin lines) at 750 (blue) and 800 °C (green).

## 4.3 Used catalyst materials characterization


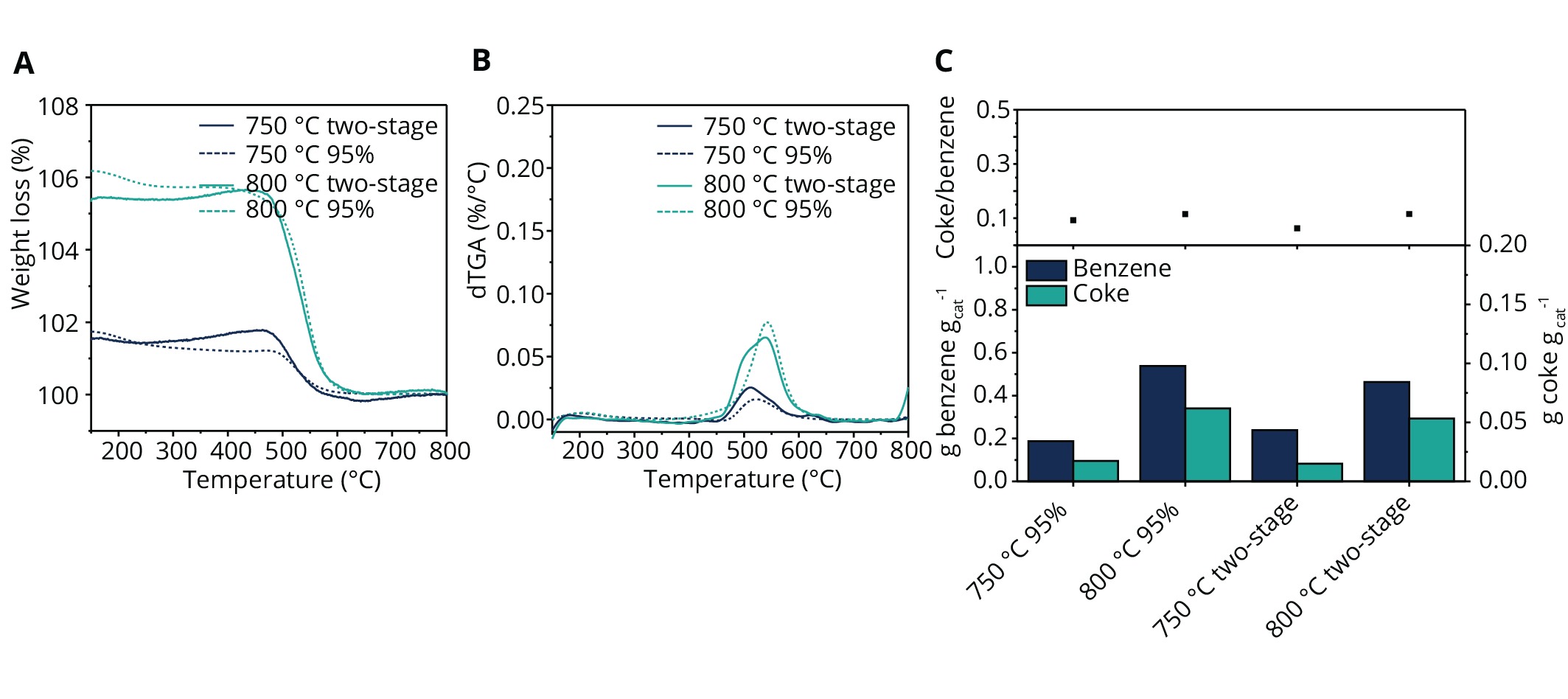


**Figure S6** Thermogravimetric analysis (TGA) results for coke analysis of the spent Mo/ZSM-5 catalysts after 10 h reaction in the two-stage reaction (solid line) and 95 % CH_4_ yield MDA simulation (CH_4_:CO_2_:H_2_:N_2_ = 18.62:0.98:3.92:2, dotted line) at 750 (blue) and 800 °C (green). (**A**) Weight loss as a function of temperature (**B**) derivative TGA curves (**C**) coke amounts as determined from the TGA results compared to the amount of benzene produced during the different catalytic experiments.


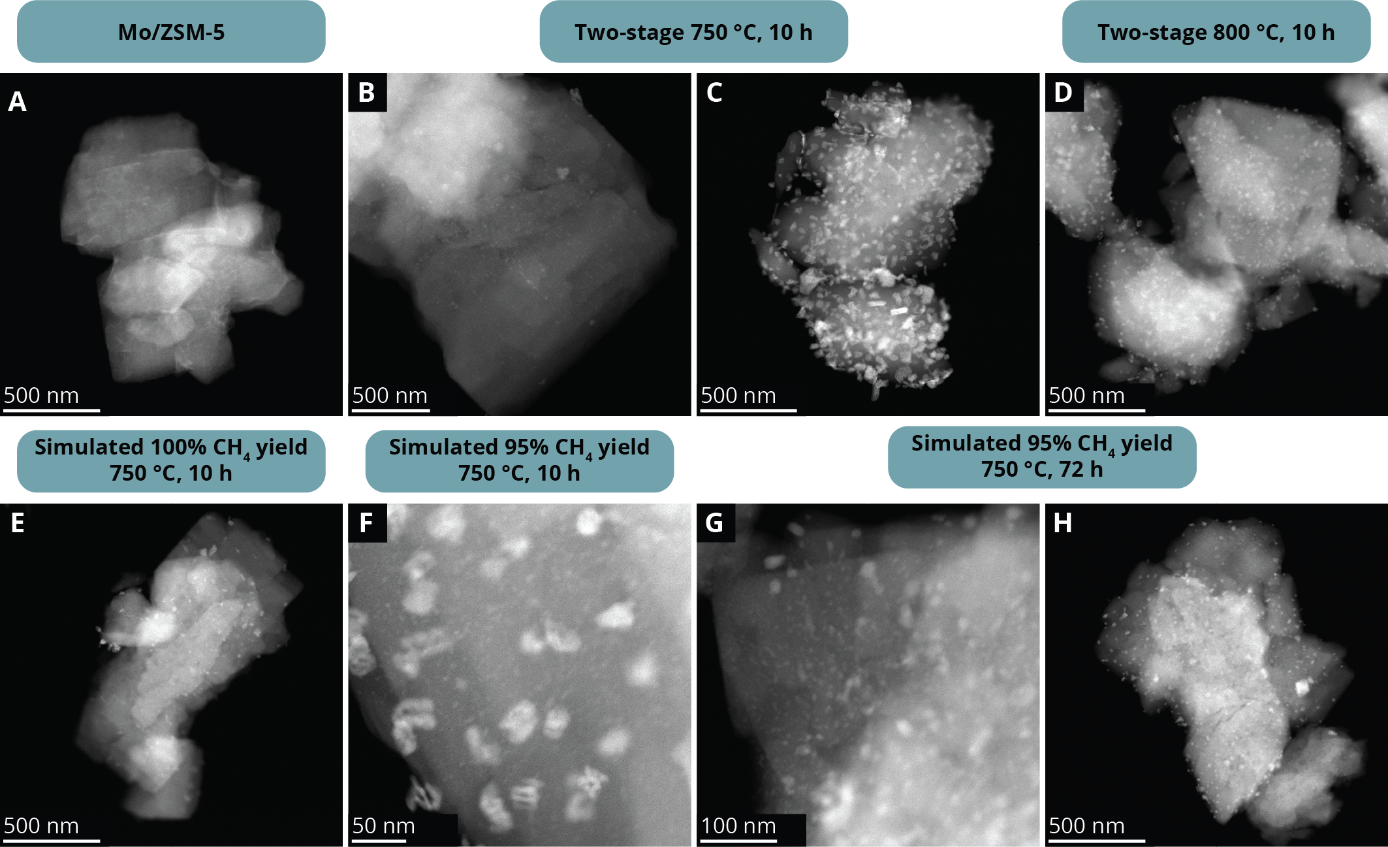


**Figure S7** High-angle annular dark-field scanning transmission electron microscopy (HAADF-STEM) images of fresh (**A**) and used Mo/ZSM-5 catalysts after either the two-stage conversion experiments at both 750 (**B,C**) and 800 °C (**D**) and after MDA experiments at 750 °C using a simulated CH_4_ yield of 100% (**E**) or 95% (both 10h (**F**) and 72h (**G,H**)).

# Thermodynamic calculations


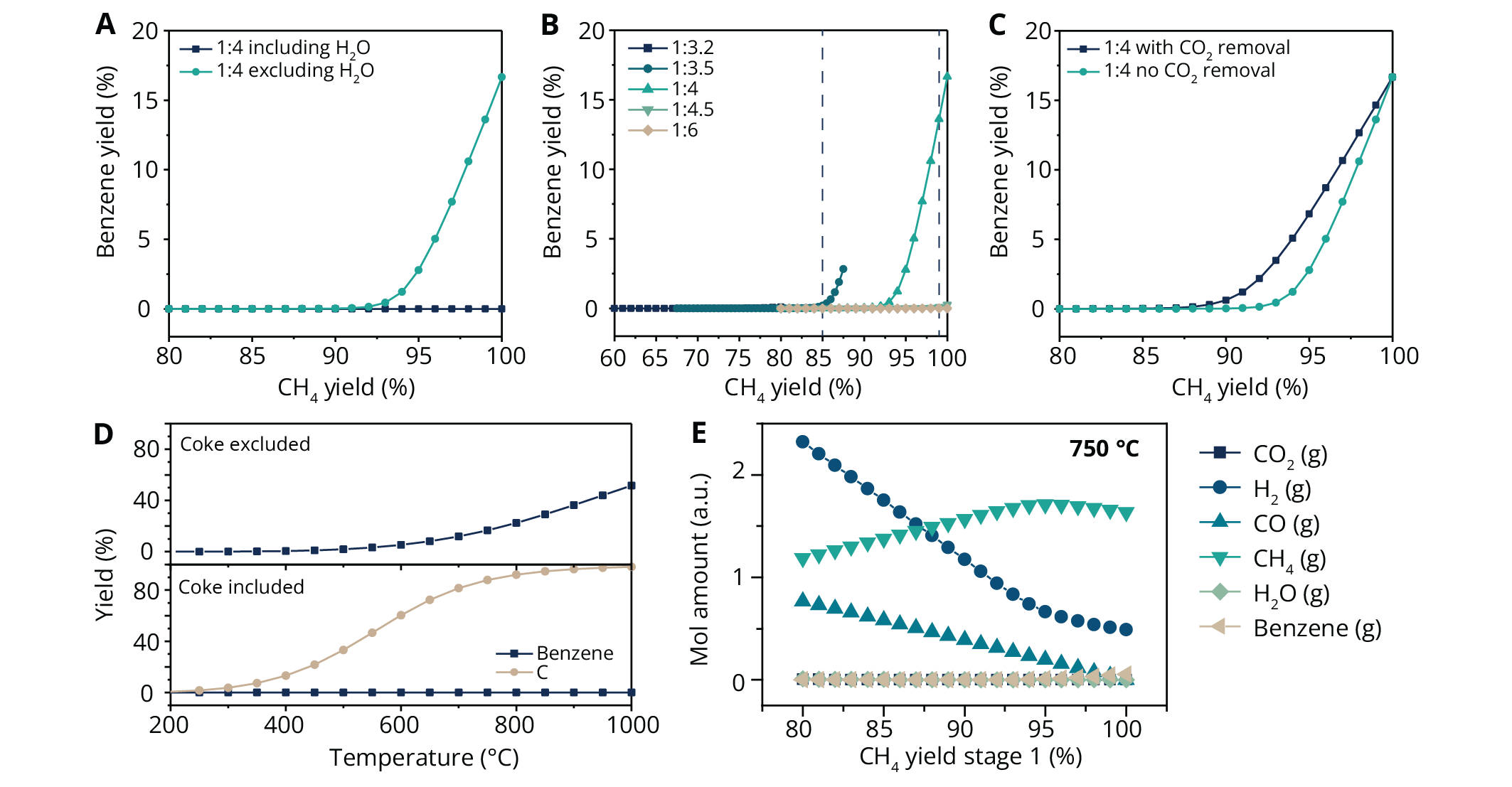
**Figure S8** Thermodynamic equilibrium calculations of benzene yield during the methane dehydroaromatization (MDA) reaction (**A**) at 750 °C as a function of CH_4_ yield in stage 1 using CO_2_:H_2_ in 1:4, when including (blue) and excluding (green) the H_2_O formed in the CO_2_ hydrogenation reaction, (**B**) at 750 °C as a function of CH_4_ yield of stage 1, where the CO_2_ and H_2_ reactants in stage 1 are fed in a ratio of 1:3.2, 1,3.5, 1:4, 1:4.5 or 1:6. The dashed lines indicate the achieved methane yield when varying the H_2_ supply to either a 1:3.5 or 1:4.5 CO_2_:H_2_ ratio over a Ni/TiO_2_ catalyst at 400 °C and 20 barg (**Figure 4B**) (**C**) at 750 °C as a function of CH_4_ yield in stage 1 using CO_2_:H_2_ ratio in 1:4 with CO_2_ removal (blue) and excluding CO_2_ removal (green), (**D**) as a function of temperature excluding (top) and including (bottom) the formation of coke, (**E**) at 750 °C as a function of CH_4_ yield from stage 1, showcasing the formation of CO due to the competitive dry methane reforming reaction at increasing CO_2_ concentration.

# Two-Stage Simulation Experiments

## 7.1 Catalytic performance in the MDA reaction


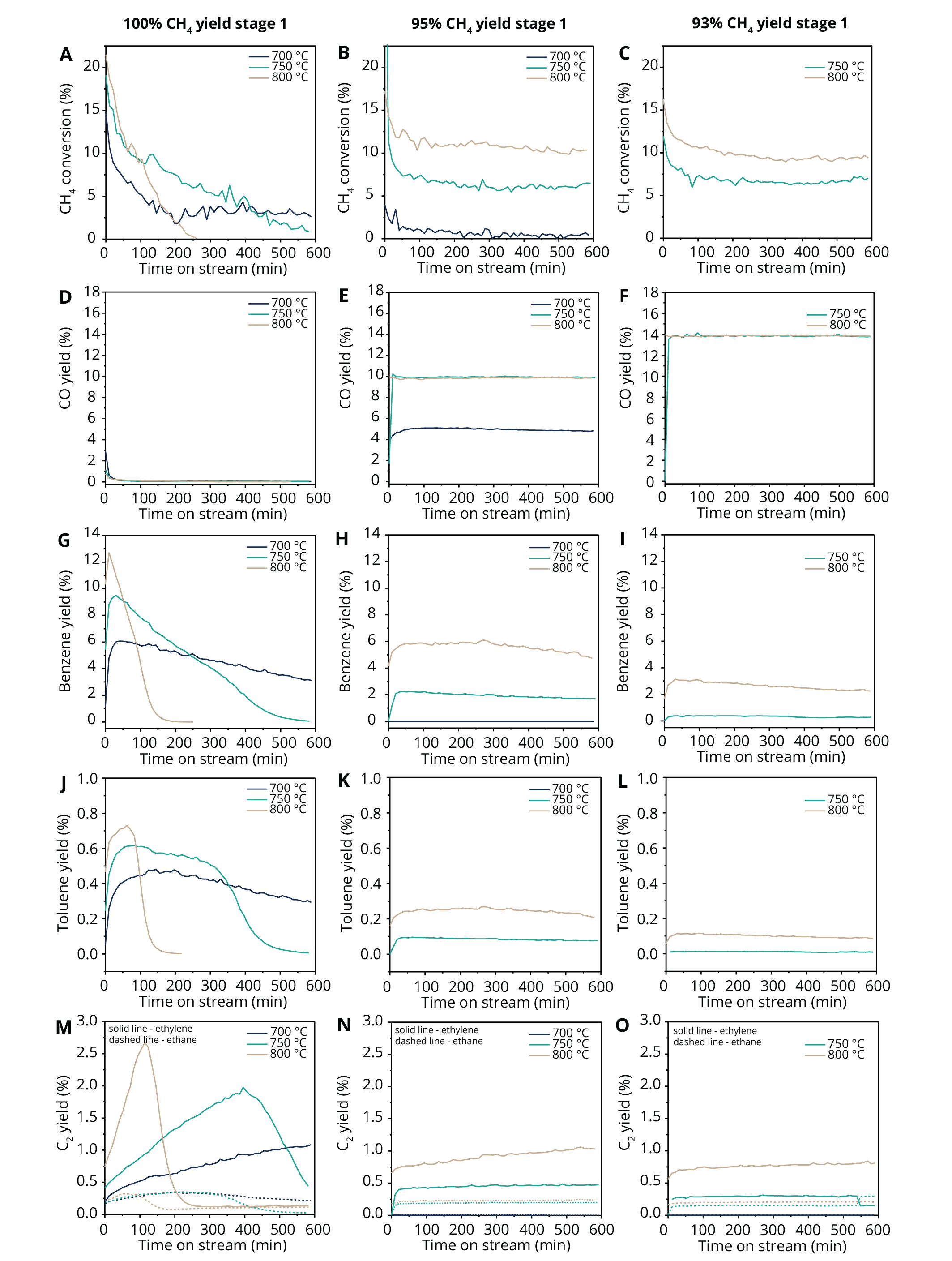


**Figure S9** Methane conversion (**A–C**) and product yields based on CH_4_ input (**D–O**) obtained from catalytic performance testing of the methane dehydroaromatization (MDA) reaction at 700, 750, and 800 °C using a reaction mixture that would be obtained from the CO_2_ methanation reaction in case of 100 (CH_4_:N_2_ = 19.6:2), 95 (CH_4_:CO_2_:H_2_:N_2_ = 18.62:0.98:3.92:2), and 93 % (CH_4_:CO_2_:H_2_:N_2_ = 18.23:1.37:5.49:2) CH_4_ yield.

## 7.2 Operando UV-Vis diffuse reflectance spectroscopy


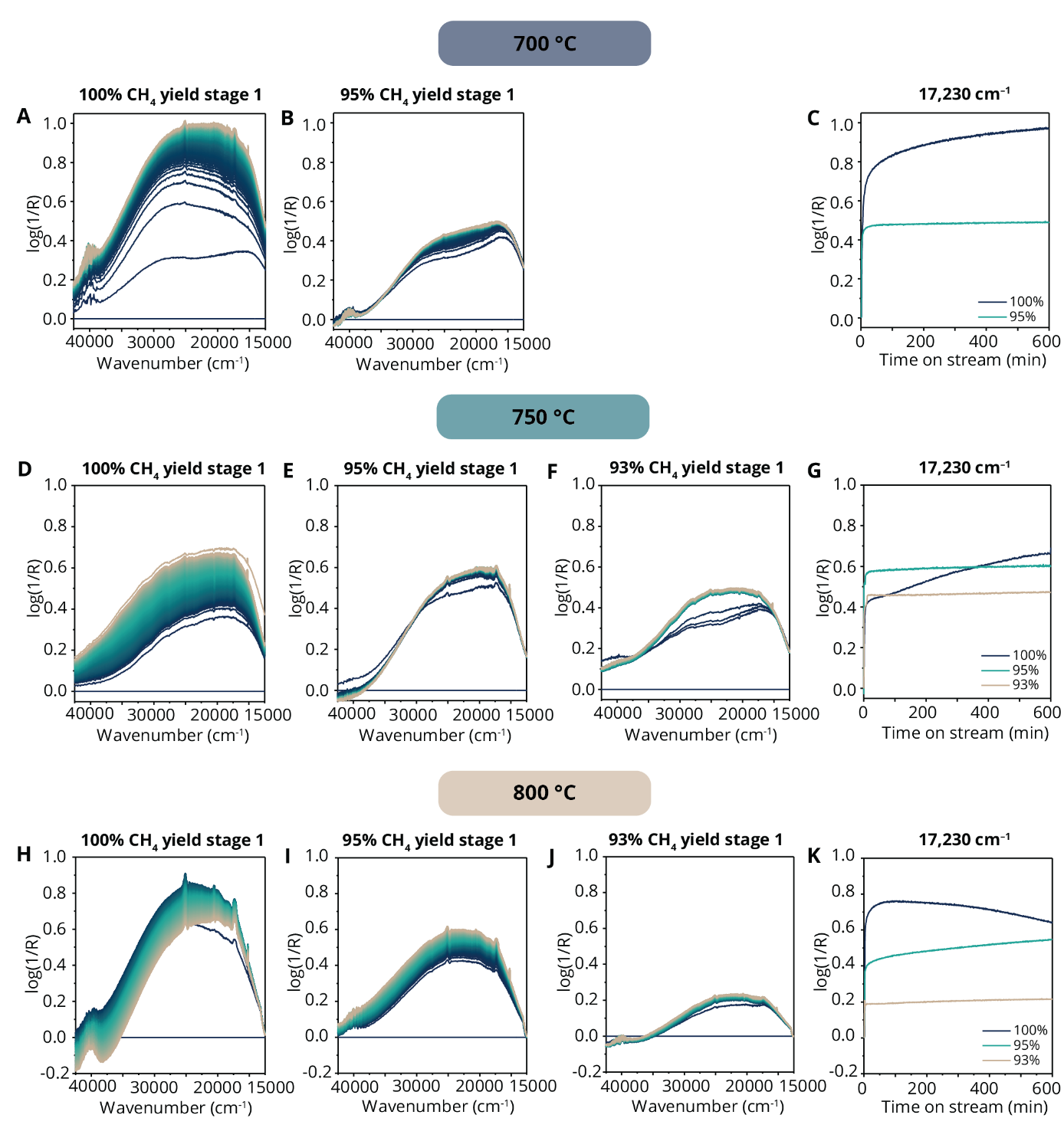


**Figure S10** Operando UV-Vis diffuse reflectance spectra recorded during methane dehydroaromatization (MDA) reaction tests when using simulated gas feeds. The figures show the spectra recorded during MDA simulating 100 (CH_4_:N_2_ = 19.6:2), 95 (CH_4_:CO_2_:H_2_:N_2_ = 18.62:0.98:3.92:2), and 93 % (CH_4_:CO_2_:H_2_:N_2_ = 18.23:1.37:5.49:2) CH_4_ yield. from stage 1 at 700, 750 and 800 °C, and the corresponding log(1/R) at 17,230 cm^–1^ over time is plotted in panels **D**, **H**, **L**.

## 7.3 Used catalysts materials characterization


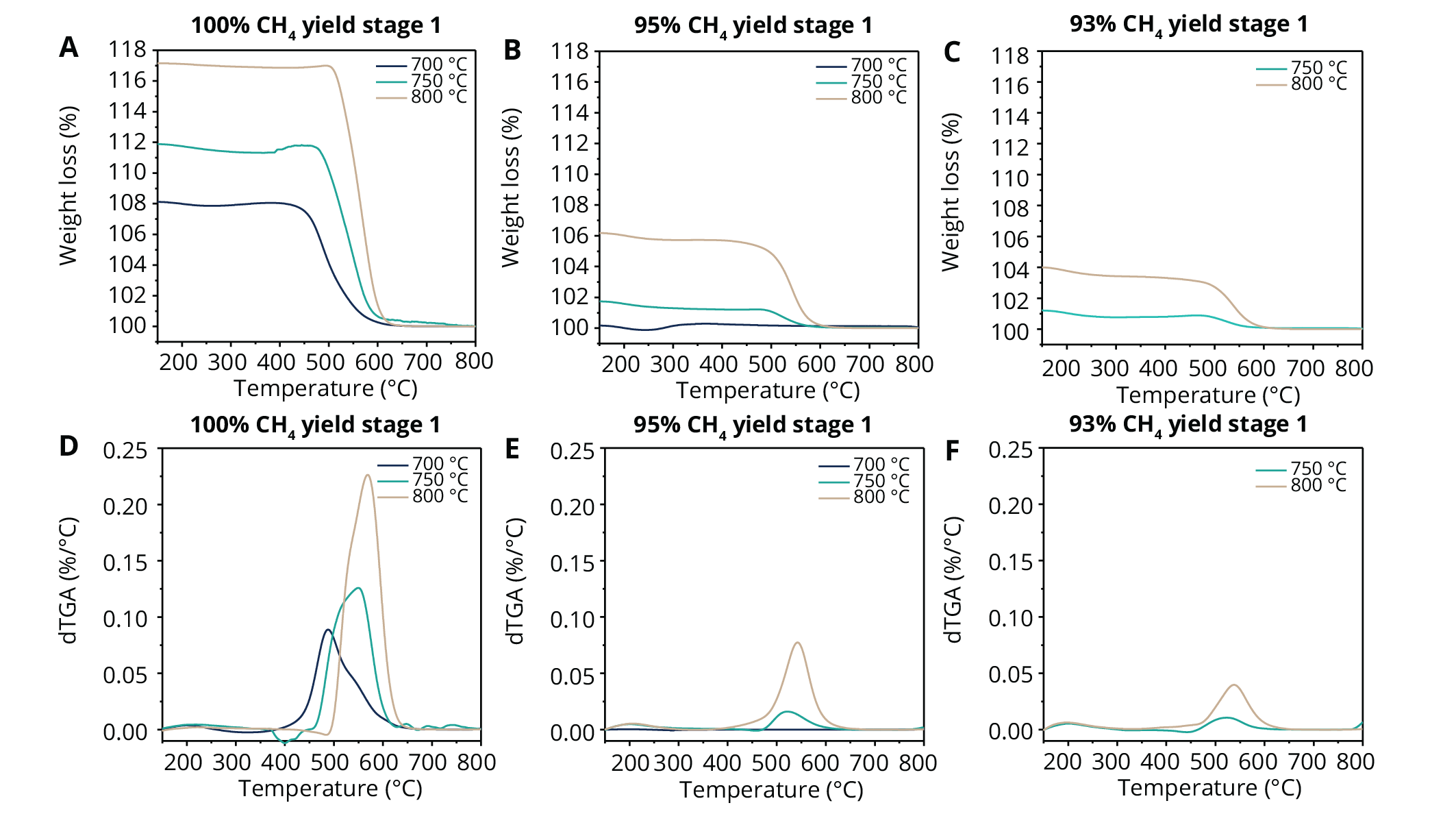


**Figure S11** Coke analysis of the spent 5 wt. % Mo/ZSM-5 catalysts after 10 h of reaction at 700, 750, and 800 °C, as measured with thermogravimetric analysis (TGA). The weight loss curves and the derivative TGA profiles are shown respectively for (**A,D**) 100 % CH_4_ yield (CH_4_:N_2_ = 19.6:2), (**B,E**) 95 % CH_4_ yield (CH_4_:CO_2_:H_2_:N_2_ = 18.62:0.98:3.92:2), and (**C,F**) 93 % CH_4_ yield (CH_4_:CO_2_:H_2_:N_2_ = 18.23:1.37:5.49:2).


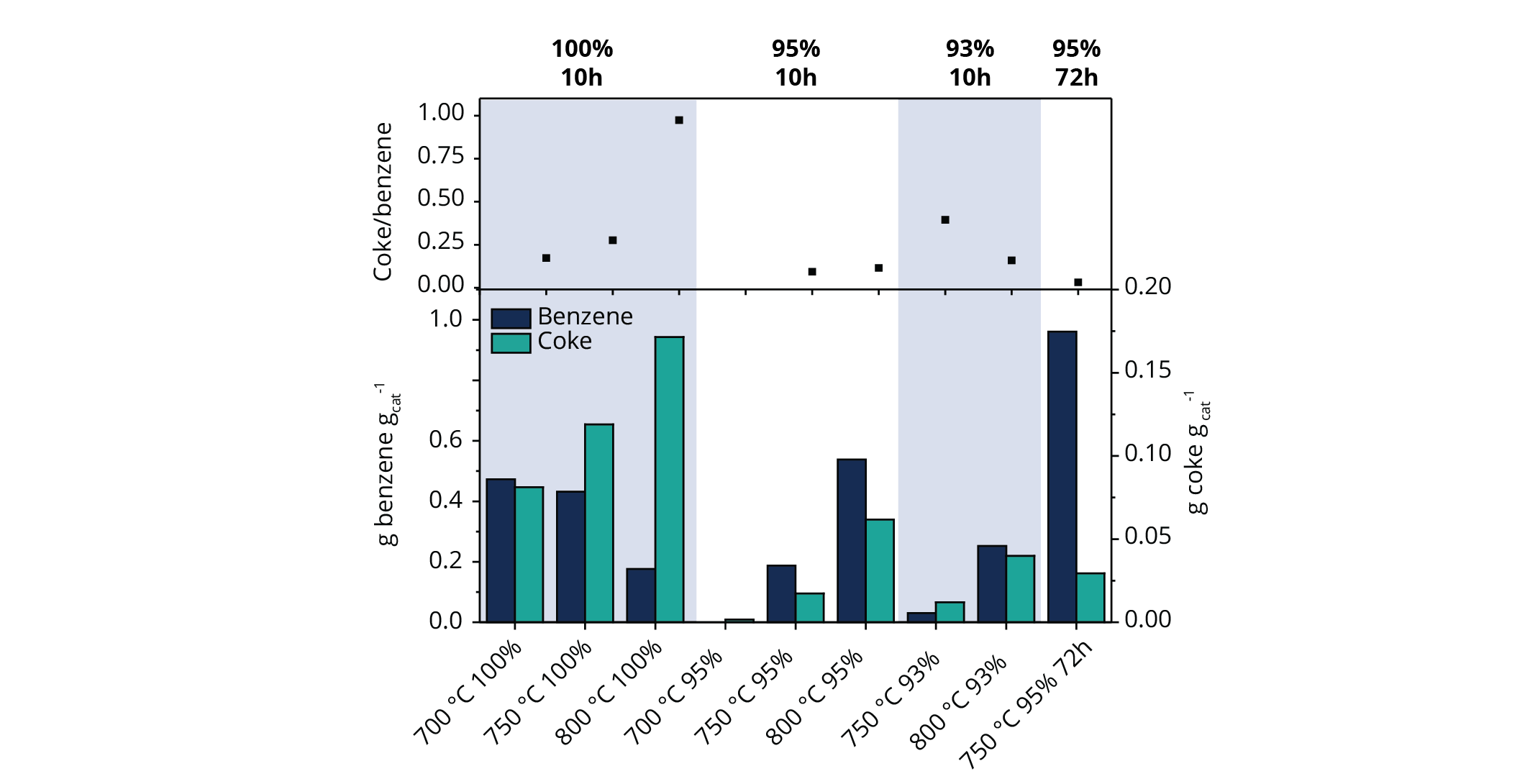


**Figure S12** Coke amounts as determined with thermogravimetric analysis (TGA) for the MDA simulation experiments, compared to the cumulative benzene production during the corresponding catalytic tests.

# Back-of-the-envelope calculations for scale of the process

For the calculations presented in **Figure 4**, we have used the CO_2_ emissions data for the Tata Steel IJmuiden site in the Netherlands from scope 1 & 2 in the Tata Steel Sustainability report 2022/2023^[13]^ (**Table S5**). For the total CO_2_ emissions of the Netherlands in 2023 we have used data provided by the Dutch Emission Authority.^[14]^ For the required H_2_ to run the CO_2_ methanation reaction, we assumed a CO_2_:H_2_ molar ratio of 1:4, and for the benzene production via MDA, we assumed a total benzene yield from CO_2_ of 5 %. **Table S6** gives a summary of the CO_2_ emissions and possible benzene yield. **Table S7** shows the fuel consumption of Air France-KLM in 2023, provided by statista.com.^[15]^ As current jet fuels contain 4–25 % aromatics,^[16–18]^ we assumed to replace 10 % of the current jet fuel consumption of Air France-KLM by synthetic benzene. **Table S8** shows the benzene production of our lab-scale system assuming no deactivation over time. These numbers were used in our calculations of the scale-up of our system up to provide the required amounts of catalyst for the total conversion of CO_2_ from the Tata Steel IJmuiden site in the Netherlands or the Netherlands (**Table S9**). Although a full scale-up of the MDA reaction is not yet operational, efforts are being made to bring this reaction to a bigger scale.^[19–21]^

**Table S5** CO_2_ emissions data for the Tata Steel IJmuiden site in the Netherlands from scope 1 & 2 in the Tata Steel Sustainability report 2022/2023.^[13]^

| Tata Steel IJmuiden 2023 | CO_2_ emissions (Mton) |
| --- | --- |
| Tata steel | 5.60 |
| Vattenfall | 5.46 |
| Linde | 0.000007 |
| DE* (scope 1) | 0.10 |
| DE (scope 2) | 0.038 |
|  |  |
| Total | **11.2**^[13]^ |

* Downstream Europe sites

**Table S6** Estimate of benzene yield from Tata Steel IJmuiden site in the Netherlands or Netherlands based on the respective CO_2_ emissions and a 5 % benzene yield.

| Tata Steel IJmuiden 2023 | CO_2_ emissions (Mton) |  | CO_2_ emissions (NL)  2023 (Mton) |  | H_2_ required IJmuiden site* (Mton) | H_2_ required (NL)*  (Mton) |  | Benzene produced IJmuiden site (Mton)** | Benzene produced (NL)  (Mton)** |
| --- | --- | --- | --- | --- | --- | --- | --- | --- | --- |
| Total | **11.2**^[13]^ |  | **58.9**^[14]^ |  | **2.1** | **11** |  | **0.17** | **0.87** |

* Assuming molar 1:4 ratio of CO_2_:H_2_

** Assuming 5 % of the CO_2_ molecules get converted into benzene

**Table S7** Estimate of how much aromatics could be provided for the fleet of Air France-KLM in 2023.

| Fuel consumption of Air France-KLM 2023 (Mton) | Aromatics needed* |  |  | Percentage of benzene that IJmuiden CO_2_ emissions can provide (%) | Percentage of benzene that NL emissions can provide (%) |
| --- | --- | --- | --- | --- | --- |
| 7.81^[15]^ | 0.78 |  |  | 21 | 112 |

* Assuming 10 wt.% of total fuel is synthetic benzene

**Table S8** Details about the thermo-catalytic two-stage process, as presented in this work.

| CO_2_ flow  (mL/min) | Benzene production* |  | Catalyst bed 1 (ton) | Catalyst bed 2 (ton) |
| --- | --- | --- | --- | --- |
| 19.6 | 0.98 mL/min |  | 5 * 10^–7^ | 6*10^–7^ |
|  | 4.0 *10^–5^ mol/min** |  |  |  |
|  | 3.1*10^–15^ Mton/min |  |  |  |

* Assuming 5 % CO_2_ gets converted into benzene

** Calculated via the ideal gas law, 298 K, 1 atm.

**Table S9** Catalyst required for the conversion of all CO_2_ emissions of the Tata Steel IJmuiden site in the Netherlands in 2023 and all CO_2_ emissions of the Netherlands based on the catalyst volume and efficiencies, as presented in this work.

| Catalyst required  for the IJmuiden site | | | |  | Catalyst required  for the Netherlands | | |
| --- | --- | --- | --- | --- | --- | --- | --- |
| Ni catalyst (ton) | **Zeolite catalyst**  **(ton)** |  | **Benzene production** |  | **Ni catalyst (ton)** | **Zeolite catalyst**  **(ton)** | **Benzene production** |
| 5 * 10^–7^ | 6 * 10^–7^ |  | 3.1*10^–15^ Mton/min |  | 5 * 10^–7^ | 6 * 10^–7^ | 3.1*10^–15^ Mton/min |
| 50.3 | 60.6 |  | 0.17 Mton/year |  | 265 | 318 | 0.87 Mton/year |

#

# References

[1] C. Vogt, M. Monai, G. J. Kramer, B. M. Weckhuysen, "The Renaissance of the Sabatier Reaction and its applications on Earth and in Space" *Nat. Catal.* **2019**, *2*, 188–197.

[2] J. Liu, C. Li, F. Wang, S. He, H. Chen, Y. Zhao, M. Wei, D. G. Evans, X. Duan, "Enhanced low‑temperature activity of CO₂ methanation over highly‑dispersed Ni/TiO₂ catalyst" *Catal. Sci. Technol.* **2013**, *3*, 2627–2633.

[3] C. Vogt, M. Monai, E. B. Sterk, J. Palle, A. E. M. Melcherts, B. Zijlstra, E. Groeneveld, P. H. Berben, J. M. Boereboom, E. J. M. Hensen, F. Meirer, I. A. W. Filot, B. M. Weckhuysen, "Understanding carbon dioxide activation and carbon–carbon coupling over nickel" *Nat. Commun.* **2019**, *10*, 5330.

[4] M. Monai, K. Jenkinson, A. E. M. Melcherts, J. N. Louwen, E. A. Irmak, S. Van Aert, T. Altantzis, C. Vogt, W. van der Stam, T. Duchoň, B. Šmíd, E. Groeneveld, P. Berben, S. Bals, B. M. Weckhuysen, “Restructuring of titanium oxide overlayers over nickel nanoparticles during catalysis” *Science* **2023**, *380*, 644–651.

[5] Wang, L.; Tao, L.; Xie, M.; Xu, G.; Huang, J.; Xu, Y. Dehydrogenation and Aromatization of Methane under Non-Oxidizing Conditions. *Catal. Lett.* **1993**, *21*, 35–41.

[6] Kosinov, N.; Hensen, E. J. Reactivity, Selectivity, and Stability of Zeolite-Based Catalysts for Methane Dehydroaromatization. *Adv. Mater.* **2020**, *32*, 2002565.

[7] Xu, Y.; Liu, S.; Guo, X.; Wang, L.; Xie, M. Methane Activation without Using Oxidants over Mo/HZSM-5 Zeolite Catalysts. *Catal. Lett.* **1994**, *30*, 135–149.

[8] Chen, L. Y.; Lin, L.; Xu, Z.; Li, X.; Zhang, T. Dehydro-Oligomerization of Methane to Ethylene and Aromatics over Molybdenum/HZSM-5 Catalyst. *J. Catal.* **1995**, *157*, 190–200.

[9] Zhang, C. L.; Li, S.; Yuan, Y.; Zhang, W. X.; Wu, T. H.; Lin, L. W. Aromatization of Methane in the Absence of Oxygen over Mo-Based Catalysts Supported on Different Types of Zeolites. *Catal. Lett*. **1998**, *56*, 207–213.

[10] Wong, S.-T.; Xu, Y.; Liu, W.; Wang, L.; Guo, X. Methane Activation without Using Oxidants over Supported Mo Catalysts. *Appl. Catal. A Gen.* **1996**, *136*, 7–17.

[11] Kosinov, N.; Coumans, F. J. A. G.; Uslamin, E. A.; Wijpkema, A. S. G.; Mezari, B.; Hensen, E. J. M. Methane Dehydroaromatization by Mo/HZSM-5: Mono-or Bifunctional Catalysis? *ACS Catal*. **2017**, *7*, 520–529.

[12] Lezcano-González, I.; Oord, R.; Rovezzi, M.; Glatzel, P.; Botchway, S. W.; Weckhuysen, B. M.; Beale, A. M. Molybdenum Speciation and Its Impact on Catalytic Activity during Methane Dehydroaromatization in Zeolite ZSM-5 as Revealed by Operando X-Ray Methods. *Angew. Chem. Int. Ed*. **2016**, *55*, 5215–5219

[13]  Tata Steel Nederland, "Tata Steel Sustainability Report 2022/2023" can be found under [www.tatasteelnederland.com/sites/default/files/tata-steel-sustainability-report-2022-2023_0.pdf](http://www.tatasteelnederland.com/sites/default/files/tata-steel-sustainability-report-2022-2023_0.pdf).

[14]  Dutch Emissions Authority, "Emissiecijfers 2021‑2023" can be found under https://www.emissieautoriteit.nl/documenten/publicatie/2024/04/15/emissiecijfers-2021-2023 **2024**.

[15]  ”Fuel consumption of Air France- KLM from 2019 to 2023 (in 1,000 metric tons), by type.” Can be found under <https://www.statista.com/statistics/1421458/air-france-klm-fuel-consumption-by-type/>.

[16]  J. Holladay, Z. Abdullah, J. Heyne, "Sustainable Aviation Fuel: Review of Technical Pathways Report" **2020.**

[17]  I. Zahid, M. H. Nazir, K. Chiang, F. Christo, M. Ameen, "Current outlook on sustainable feedstocks and processes for sustainable aviation fuel production" *Curr. Opin. Green Sustain. Chem.* **2024**, *49*, 100959.

[18]  D. Zakgeym, N. Bottke, K. Braunsmann, A. Higelin, M. Schwab, T. Kimpel, H. Kömpel, T. Streich, "Designing a Sustainable Aviation Fuel (SAF) Production Process from Methanol: Towards a Greener Future in the Aviation Sector" *Nitrogen + Syngas Expoconference,* presented at Barcelona 10-12 February 2025**.**

[19]  C. Mevawala, X. Bai, J. Hu, D. Bhattacharyya, "Plant‑wide modeling and techno‑economic analysis of a direct non‑oxidative methane dehydroaromatization process via conventional and microwave‑assisted catalysis" *Appl. Energy* **2023**, *336*, 120795.

[20]  Z. G. Zhang, "Process, reactor and catalyst design: Towards application of direct conversion of methane to aromatics under nonoxidative conditions" *Carbon Resour. Convers.* **2019**, *2*, 157–174.

[21] I. Julian, C. M. Pedersen, A. B. Jensen, A. K. Baden, J. L. Hueso, A. V. Friderichsen, H. Birkedal, R. Mallada, J. Santamaria, "From bench scale to pilot plant: A 150× scaled‑up configuration of a microwave‑driven structured reactor for methane dehydroaromatization" *Catal. Today* **2022**, *383*, 21–30.
